# Recombination-driven generation of the largest pathogen repository of antigen variants in the protozoan *Trypanosoma cruzi*

**DOI:** 10.1186/s12864-016-3037-z

**Published:** 2016-09-13

**Authors:** D. Brent Weatherly, Duo Peng, Rick L. Tarleton

**Affiliations:** 1 Center for Tropical and Emerging Global Diseases, Institute of Bioinformatics and Department of Cellular Biology, University of Georgia, Athens, GA 30602 USA; 2 Center for Complex Carbohydrate Research, University of Georgia, Athens, GA 30602 USA

**Keywords:** Chagas disease, Trans-sialidase, *Trypanosoma cruzi*, Immune evasion, Recombination, Antigen variants

## Abstract

**Background:**

The protozoan parasite *Trypanosoma cruzi,* causative agent of Chagas disease, depends upon a cell surface-expressed *trans*-sialidase (ts) to avoid activation of complement-mediated lysis and to enhance intracellular invasion. However these functions alone fail to account for the size of this gene family in *T. cruzi*, especially considering that most of these genes encode proteins lacking ts enzyme activity*.* Previous whole genome sequencing of the CL Brener clone of *T. cruzi* identified ~1400 ts variants, but left many partially assembled sequences unannotated.

**Results:**

In the current study we reevaluated the *trans*-sialidase-like sequences in this reference strain, identifying an additional 1779 full-length and partial ts genes with their important features annotated, and confirming the expression of previously annotated “pseudogenes” and newly annotated ts family members. Multiple EM for Motif Elicitation (MEME) analysis allowed us to generate a model *T. cruzi* ts (TcTS) based upon the most conserved motif patterns and demonstrated that a common motif order is highly conserved among ts family members. Using a newly developed pipeline for the analysis of recombination within large gene families, we further demonstrate that TcTS family members are undergoing frequent recombination, generating new variants from the thousands of functional and non-functional ts gene segments but retaining the overall structure of the core TcTS family members.

**Conclusions:**

The number and variety as well as high recombination frequency of TcTS family members supports strong evolutionary pressure, probably exerted by immune selection, for continued variation in ts sequences in *T. cruzi*, and thus for a unique immune evasion mechanism for the large ts gene family.

**Electronic supplementary material:**

The online version of this article (doi:10.1186/s12864-016-3037-z) contains supplementary material, which is available to authorized users.

## Background

The first complete genome sequence for the protozoan parasite *T. cruzi* was reported in 2005, along with the genomes of the related parasites *Trypanosoma brucei* and *Leishmania major*. Together, these parasites are the cause of substantial morbidity and mortality and thus are the focus of continued research to prevent and treat the diseases that they cause.

One of the unique features of the *T. cruzi* genome is the abundance of large gene families, groups of homologous and functionally related genes that exist in multiple loci across the genome. Greater than 25 % of the genome of *T. cruzi* is comprised of families of genes with at least 20 members [1]. In contrast, the gene family composition for *T. brucei* and *L. major* is approximately 6 and 12 % respectively [2, 3]. Furthermore, three of these gene families in *T. cruzi,* the *trans*-sialidases, mucins and mucin-associated surface proteins (MASPs) each encode more than 500 unique surface membrane proteins. One of the purposes of this study is to more fully inventory the largest of these families, the *trans*-sialidases, and to determine the mechanism of their massive diversification in *T. cruzi*.

The *T. cruzi trans*-sialidases (TcTS) are glycosylphosphatidylinositol-anchored surface proteins produced by trypomastigotes and amastigotes of *T. cruzi*. A minority (as few as 20 [4, 5]) of the TcTS genes encode enzymatically active proteins that transfer sialyl residues from host donor molecules to *T. cruzi* glycoproteins and glycolipids. Because *T. cruzi* and other trypanosomatids are unable to synthesize sialic acid *de novo*, this transferase activity is the only mechanism for sialylation of parasite surface molecules and is crucial to parasite infectivity and survival [6]. The purpose of the vast majority of TcTS genes which apparently lack *trans*-sialidase activity and the reason for the development and expansion of this and other gene families selectively in *T. cruzi* is not understood. One hypothesis is that multiple copies of similar genes might serve an immune evasion function for *T. cruzi* by presenting to the immune system a multitude of different and potentially constantly varying surface molecules. It is further postulated that these gene families have expanded in response to immune pressure, presumably by duplication, recombination and mutation of the ancestral founding family members.

While the genome provides ample evidence of large gene families in *T. cruzi*, it must be noted that the presence of large numbers of homologous genes and other repetitive elements also hindered genome assembly and a complete inventory of gene family members. In fact, the genome was not initially fully assembled into chromosomes but was rather published as 32,000 contigs (only 4000 of which were annotated to contain coding sequences), some of which were mapped to ~600 scaffolds. Given that there are over 27,000 unannotated contigs (albeit most of these are quite short) the question arises as to what these contigs contain. Focusing specifically on the TcTS family, we have asked if there are additional, previously unannotated TcTS genes in the genome.

In this work we describe the method to specifically identify all *trans*-sialidase-like sequences in the reference *T. cruzi* CL Brener genome, raising the total number from the 1430 initially annotated to 3209. Next, we verified and adjusted the predicted bounds of the annotated TcTS genes (i.e. start/stop coordinates), including among these TcTS nucleotide sequences that contain in-frame stop codons. Motif analysis using Multiple EM for Motif Elicitation (MEME) provided a means to compare the structure of all 3209 TcTS sequences and to generate a model TcTS based upon the most conserved motif patterns. Lastly, we show that TcTS family members have been undergoing recombination, thus generating new variants from the thousands of pieces, while retaining the overall structure of the core TcTS family members.

## Results

### Identification of previously unannotated TcTS sequences

Prior to beginning analysis of the TcTS family, we first made sure that we had identified the complete complement of TcTS genes for the reference CL Brener clone of *T. cruzi*. Previous work by us (unpublished) and others [7] indicates that the number of sequenced TcTS genes is underestimated, as evidenced by merging of large numbers of whole genome shotgun (WGS) reads into relatively few annotated genes. Additionally, proteome analysis has shown that *T. cruzi* peptides map to sequence reads that are not assigned to annotated proteins [5]. Furthermore, a preliminary remapping of WGS reads selected because of their homology to TcTS gene sequences to the assembled genome revealed that a large number of these TcTS-like reads mapped to regions with no annotated genes. Thus this work sought to determine if additional, unannotated TcTS genes might be identifiable, and if so, how these newly annotated genes compared to the existing set of previously annotated TcTS genes.

Assembly of the *T. cruzi* CL Brener genome was difficult, in part because of the hybrid nature of this parasite clone and, more importantly, because of the very high number of closely related genes that form large gene families. In our attempts to provide a more complete and accurate assembly, we have, among other things, remapped the original sequencing reads from the whole genome shotgun (WGS) to the assembled genome (Additional file 1: Figure S1). In order to separate potentially merged TcTS genes [7, 8] into their individual genes, the sequences for all 1430 annotated TcTS genes from the CL Brener genome, along with up to 1Kb flanking sequence, were BLASTed (BLASTN) [9] against all 1,131,562 wgs reads. All reads for which the best hit was to a TcTS-like region with at least 90 % sequence identity were identified, resulting in 257,824 TcTS-like reads. Subsequently, the TcTS-like reads were BLASTed against all 32,746 genome contigs to identify the regions of the genome that were most homologous to each read (min 90 % sequence identity). This read-to-contig assignment revealed 71,228 reads mapping to regions containing annotated TcTS genes and 57,411 mapping to non-TcTS genes. Given that the original source sequences included TcTS-flanking regions, these results are not surprising. However, nearly 130,000 reads mapped to regions of the genome that contained no annotated genes. Since these regions were identified from TcTS-like reads, it was postulated that they contained either unannotated TcTS genes or genes that typically flank TcTS genes (within the 1 kb bounds of the flanking sequences used in the analysis).

**Fig. 1:**
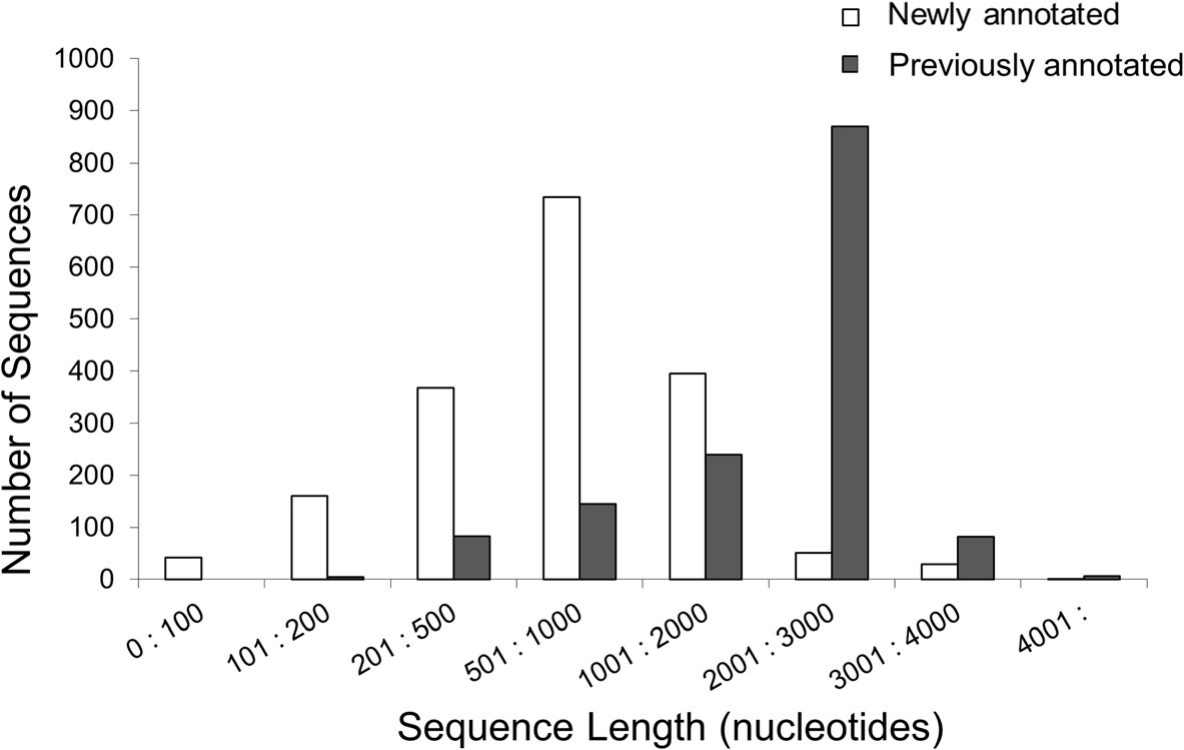
Comparison of size of previously and newly annotated TcTS genes. The DNA sequences for the TcTS genes in the annotated genome and those newly identified were binned according to sequence length in the ranges displayed and the quantity in each bin plotted for each sequence type

Using the regions of homology from the BLAST report, 9852 distinct (non-overlapping) contig regions were identified from the reads that mapped to no annotated genes. To determine which of these regions contained full or partial TcTS genes, the DNA sequences for the contig regions were extracted and BLASTed (3-frame translated BLASTX) against the 735 TcTS genes annotated as full-length (“real”; containing both signal peptide and GPI anchor motifs and whose amino acid translations resulted in no in-frame stop codons; Additional file 2: Table S1a). BLASTX was used in order to identify contiguous TcTS sequences on the contigs regardless of frame shifts. Using a minimum identity cutoff of 90 %, the contiguous regions of contigs matching the TcTS genes were identified, resulting in 2007 candidate TcTS genes. To help ensure that the new TcTS genes were sufficiently *trans*-sialidase-like, the 2007 sequences were BLASTed (BLASTP) against the entire genome to confirm that the most homologous protein for each newly identified TcTS-like sequence was an annotated *trans-*sialidase gene. From this validation step, 1780 were selected as newly identified TcTS genes. The new TcTS sequences were on average shorter than the originally annotated set of TcTS genes (some <100 bp) but also included many full or nearly full length genes (Additional file 2: Table S1b,c and Fig. 1). One hundred fourteen (114) of these newly identified TcTS genes mapped to the assembled CL Brener chromosome pairs [10] while the remaining 1662 genes mapped to contigs and scaffolds that could not be assembled into the 41 chromosomes. This contrasts with the 1062 out of 1430 previously annotated TcTS genes that mapped to the chromosomes (Additional file 2: Table S1d,e).

### Modification of TcTS coding sequences

The above described analysis more than doubled the number of identified TcTS sequences in the *T. cruzi* genome and posed the additional question of ‘what characteristics are common to these >3000 TcTS gene sequences?’, i.e. ‘what defines a TcTS protein?’. In order to address this question, we submitted the TcTS genes to three analyses and potential modifications. First, to confirm that the 5′ and 3′ bounds for all TcTS genes (both previously and newly annotated) encompassed the entire predicted coding region, 40 “model” full-length TcTS genes (Additional file 2: Table S1f) of at least 2Kb in length and containing both a signal peptide and GPI anchor motif were selected and were BLASTed (BLASTX) against the designated nucleotide sequence for all TcTS sequences (plus an additional 500 flanking bases). The predicted bounds of each TcTS sequence were then set according to its homology with these model genes. This analysis resulted in the alteration in the bounds for 207 of the 1430 previously annotated TcTS genes and the merging of two adjacent sequences (TcCLB.505267.20 and .10) into a single TcTS gene (Additional file 3: Figure S2, Additional file 2: Table S1b,c).

Second, deletions causing frame-shifts that result in stop codons are present in nearly half of the TcTS genes and were designated as “pseudogenes” in the annotated CL Brener genome. Previously, we demonstrated that a number of TcTS pseudogene-encoded proteins are detected in the *T. cruzi* proteome [5], indicating that these are real genes that probably produce a truncated TcTS product. In many cases, the DNA sequence beyond these stop sites were homologous to other TcTS gene sequences but presumably not translated into protein. However, if these sequences beyond the stop sites were at some point recombined with other TcTS genes (see below), a shift in reading frame could result in production of a TcTS protein from this sequence. Thus in order to generate a complete set of *potential* TcTS protein sequences, the TcTS sequences were examined for the presence of in-frame stop codons that might result in a truncated product, and if present, the reading frame was shifted by removing one or more bases to create a TcTS-like protein (i.e. the protein sequence beyond the stop site that was homologous to the putatively functional TcTS genes was included in the *virtual* protein sequence (Additional file 4: Figure S3)). This latter exercise also served to expand our TcTS protein set to include sequences that could have been erroneously terminated due to sequencing or assembly errors and allowed us to utilize the complete potential protein sequence information for genes previously annotated as “pseudogenes” in the analysis of proteomic data, for protein motif searches, and for examination of the products of gene recombination.

Third, to improve upon the annotation information for TcTS sequences (the initial annotations simply indicate that they are *trans-*sialidase-like and whether they are “pseudogenes”), all TcTS sequences were re-annotated based on their domain composition, length, sequence type, and whether or not their translations result in any in-frame stop codons. Among the potential features annotated in this process are a signal peptide, GPI anchor addition site, shed acute-phase antigen (SAPA) repeats, FRIP motif (xRxP), and the Asp block (SxDxGxTW) and VTVxNVxLRN motifs [4]. We provide three TcTS sequence types, where a “full length” sequence must contain both a signal peptide and GPI anchor motif, a “partial” sequence contains a signal peptide motif but no GPI anchor, and a “pseudogene” contains no signal peptide motif. Finally, if the amino acid translation of the sequence results in any stop codons, we notate that sequence with “in-frame stop” (Additional file 2: Table S1).

### Confirmation of TcTS coding sequences

To evaluate if the newly identified TcTS, most of which were not full-length genes, also encoded *bona fide* proteins, a database of all *T. cruzi* proteins (including previously annotated as well as newly annotated TcTS proteins) was created and screened with the spectra from three proteomic analyses using a 6-frame translation. This analysis identified a minimum of 156 *trans-*sialidase proteins, 70 of which were among those newly annotated as described above (Additional file 5: Table S2). This is a highly conservative estimate of the expressed repertoire due to the limitation of proteomic techniques and the difficulty of uniquely mapping small peptides to a set of highly similar proteins like those expressed by TcTS genes, as previously discussed [5]. If we exclude peptides that map to protein translations of both previously annotated and newly annotated TcTS genes, 27 newly annotated TcTS genes had unique peptide evidence of expression (“validated” in Additional file 5: Table S2a). Thus, of the 156 TcTS protein sequences identified in the proteomic analysis, almost half (45 %, 70 of 156) were new TcTS sequences, of which 38 % (27 of 70) were unambiguously identified with high-confidence. Additionally, 27 previously annotated TcTS “pseudogenes” were confirmed to yield protein products.

### Motif identification and construction of a model TcTS gene

The large size of the TcTS family made sequence alignment an untenable method for examining the common sequence features of the >3000 genes. Thus we employed the MEME tool [11] because it allowed us to identify common sequence features of the TcTS proteins and to determine the representation of these features in all members of the family. We arbitrarily set the MEME analysis to identify 100 features among the TcTS family proteins; motifs with the lowest number are the most common and most highly conserved while those closer to 100 are less so. Of the 3209 total TcTS sequences, 3176 contained at least 1 motif, and motif architectures were determined for these sequences and aligned (Additional file 6: Figure S4 and Additional file 7: Table S3).

**Fig. 2:**
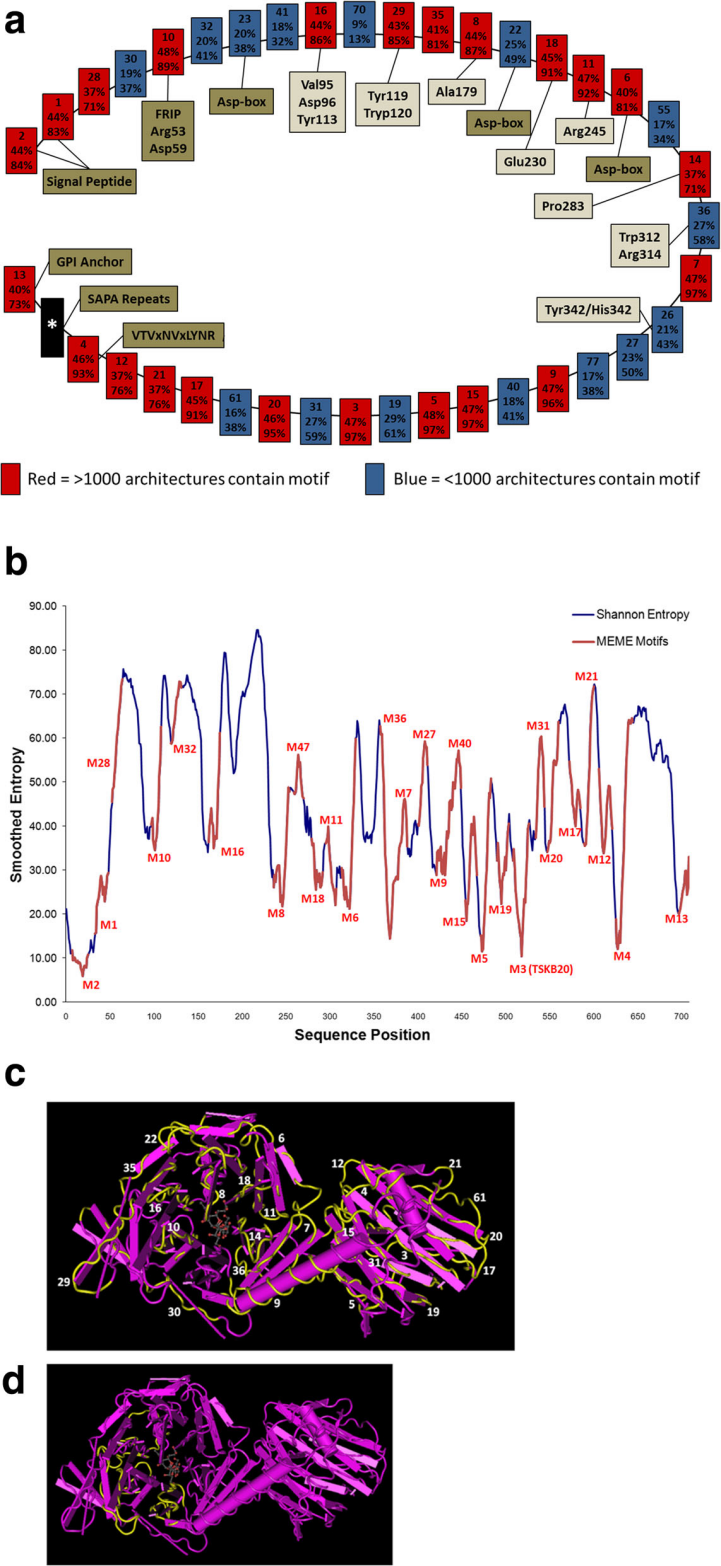
Analysis of Model TcTS Architecture. **a** The model TcTS architecture (starting with the signal peptide motifs) is depicted. Each box represents an individual motif from the MEME analysis. The top number is the motif number, the middle percentage is the representation of that motif across all TcTS sequences (including where absent), and the bottom number is the representation of that motif in TcTS sequences of length >2000b. Frequent motifs (>1000 occurrences in all TcTS sequences) are *color-coded red*, while less frequent motifs are *blue*. The highly-variable C-terminal region containing multiple motifs is represented as a *black box*. Locations of previously characterized TcTS motifs and residues are also shown. **b** An entropy analysis of TcTS sequences is shown. The smoothed entropy values for selected TcTS proteins is plotted against amino acid position. Using sequence homology of the motifs in this study to the protein sequences of the entropy study, locations of the motifs are overlaid in the chart (in *red*). Note that lower-numbered motifs are typically found in areas of low entropy. **c** The crystal structure of TcTS “1S0I_A” with motif regions highlighted and labeled and **d**) a notable region lacking in motifs (see Additional file 11: Figure S5 for diagram of motifs mapped to the crystal structure)

A model TcTS architecture was determined from the alignment by selecting the most common motif at each position (Fig. 2a). Though the model does not capture all of the variation present in the alignment, it does provide a global representation of the most common features in TcTS sequences. Not surprisingly, the most common motifs (and thus most highly conserved sequences) are in the N-terminal region containing the signal sequence (motifs 1 and 2). Other known TcTS features are Asp-boxes (motifs 23, 22, 6), FRIP (10), GPI Anchor (13), and the VTVxNVxLYNR motif (4). The C-terminal region between the VTVxNVxLYNR and GPI Anchor is variable and sometimes contains SAPA repeats. Finally, selected motifs implicated in the catalytic mechanism of TcTS were located in the motifs (10, 16, 29, 8, 18, 11, 14, 36, 26). Examination of the 3176 TcTS sequences relative to the model reveals a very high degree of conservation in the ordering of the motifs; with few exceptions, the order of motifs is maintained even in the small and thus partial TcTS sequences. At any particular position in the alignment, several different motifs may be observed across the architectures, but it is rare for a motif to be observed at multiple positions, especially for the higher-confidence (low numbered) motifs. Thus, the motif analysis of all TcTS family members in the CL Brener genome suggests a mechanism for the retention of a common TcTS protein structure despite the nucleotide diversity among the gene family members.

Not surprisingly, the most highly conserved patterns correlate with low entropy measurements (Fig. 2b). Mapping of the motifs to the crystal structure of the 1SO1_A TcTS protein [12] demonstrates the regions of higher conservation among TcTS molecules (Fig. 2c and Additional file 8: Figure S5) and reveals a large region of the catalytic domain with sparse mapping of motifs, indicating this as a more variable region among TcTS proteins (Fig. 2d). The heat map of Additional file 9: Figure S6 (and in detail in Additional file 7: Table S3, Confidence Heatmap tab) provides a global view of the degree of conservation of motifs along with the variability that provides diversity. The typical scenario shows either high conservation (model motif is frequent (red cells at top) with low entropy regions (short gray bars) and high-confidence motif-to-architecture assignments (red cells in the column)) or high diversity (blue cells at top, high gray bars, blue/purple columns). Of particular interest are columns that show seemingly contradicting data: high frequency model motif (red header cell) with relatively high entropy (high gray bar, blue/purple column) or low frequency model motifs (blue header cell) with low entropy (low gray bar, red column). An example of the former is motif 28. This region occurs after the signal peptide and is represented in a high number of sequences (71 % of the TcTS sequences >2000 bp). One interpretation of this result is the constraint in this area retains protein structure but does not maintain any specific function; so long as the protein conformation is correct the sequence is relatively free to mutate. An example of the latter case is motif 36, which displays relatively low entropy but is not highly prevalent across the architectures (58 % of TcTS sequences >2000 bp). The mapping of the motifs to the structure shows that this motif is near the central, α–helix region connecting the N-terminal and C-terminal domains (Fig. 2c). The minimum entropy among all residues in motif 36 is 0.14 and the maximum is 0.62, suggesting tight constraint at particular positions but not on the motif region as a whole.

### Phylogenetic tree of related TcTS sequences

**Fig. 3:**
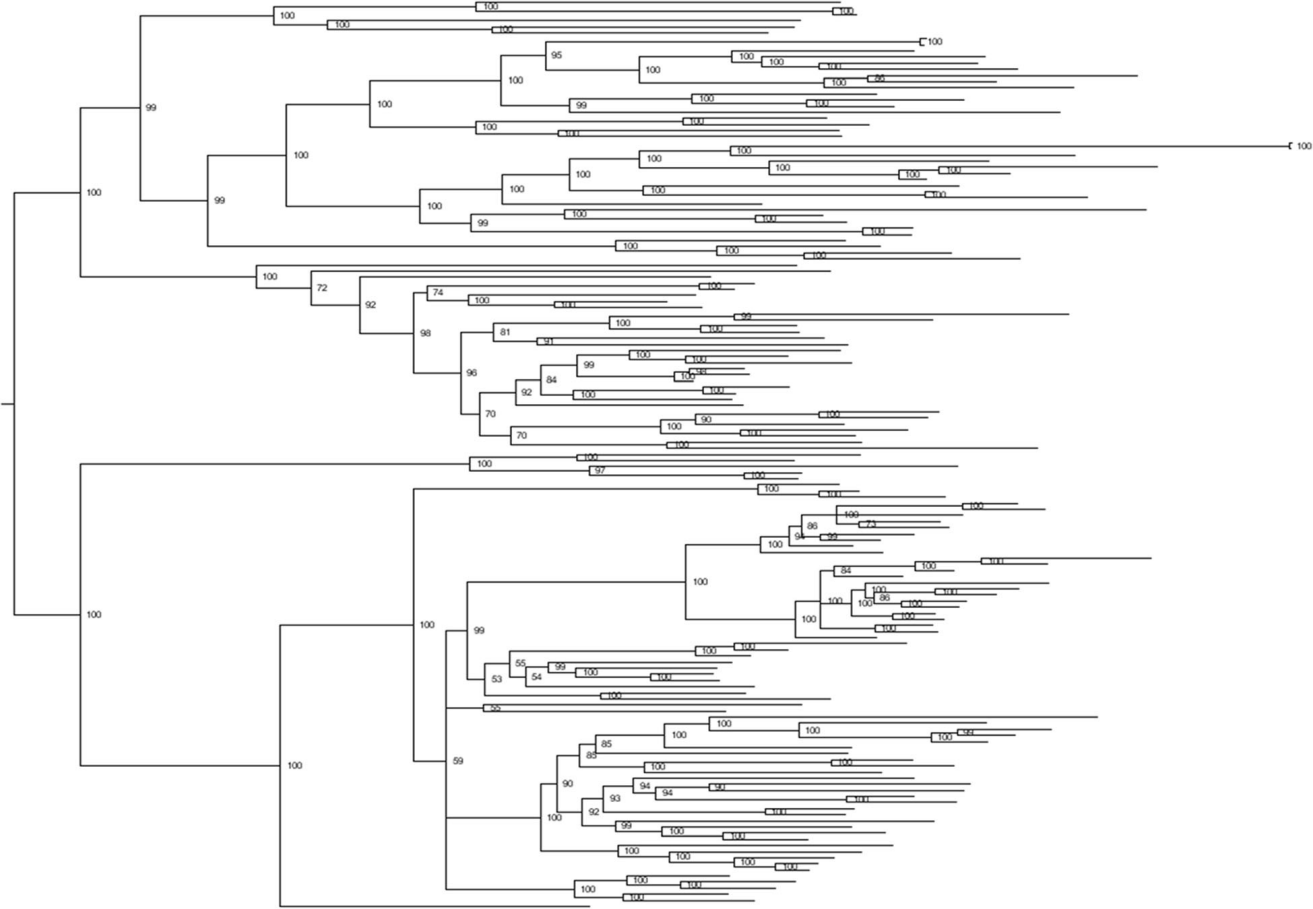
Bayesian phylogenetic tree of 149 closely related TcTS. In order to construct a phylogenetic tree from high quality alignment, sequences from 149 TcTS genes with high pairwise identity were selected. Sequences were aligned using MUSCLE algorithm, and the tree was constructed by MrBayes v3.2.1. Posterior probability values are indicated on tree nodes. Low treeness (=0.363; calculated by dividing the cumulative internal branch length by the total length of the tree) provides support for recombination among TcTS gene family members

The distribution throughout the *T. cruzi* genome of TcTS sequences of varying sizes but with a conserved structure strongly suggests a mechanism of gene duplication followed by recombination and mutation in the evolution of this large and diverse gene family. To explore this possibility further, we first examined phylogenetic trees of TcTS sequences for evidence of recombination. Due to the substantial variation in gene length and nucleotide polymorphism among the >3000 TcTS sequences (which resulted in poor sequence alignment), we performed phylogenetic tree inference on small sets of more closely related TcTS sequences identified by MEGABLAST with custom thresholds (see Materials and Methods for details). Although the potential mosaic nature of TcTS sequences could hinder accurate phylogeny assessment, we were able to estimate a well-supported bayesian tree (Fig. 3) by a lengthy and aggressive Markov chain Monte Carlo exploration in parameter space. This Bayesian phylogenetic tree has long terminal branches, indicating that either (1) evolution is accerated in all analyzed genes, since their divergence from their most recent common ancestors, or (2) disruptions in gene-independent evolution by processes such as recombination, which exchanges genetic information between genes and thus obscures ancestry. Notably, a similar tree pattern is observed for the variant surface glycoprotein (VSG) genes in *T. brucei* and *T. congolense* [13].

### Assessment of recombination events in TcTS

**Fig. 4:**
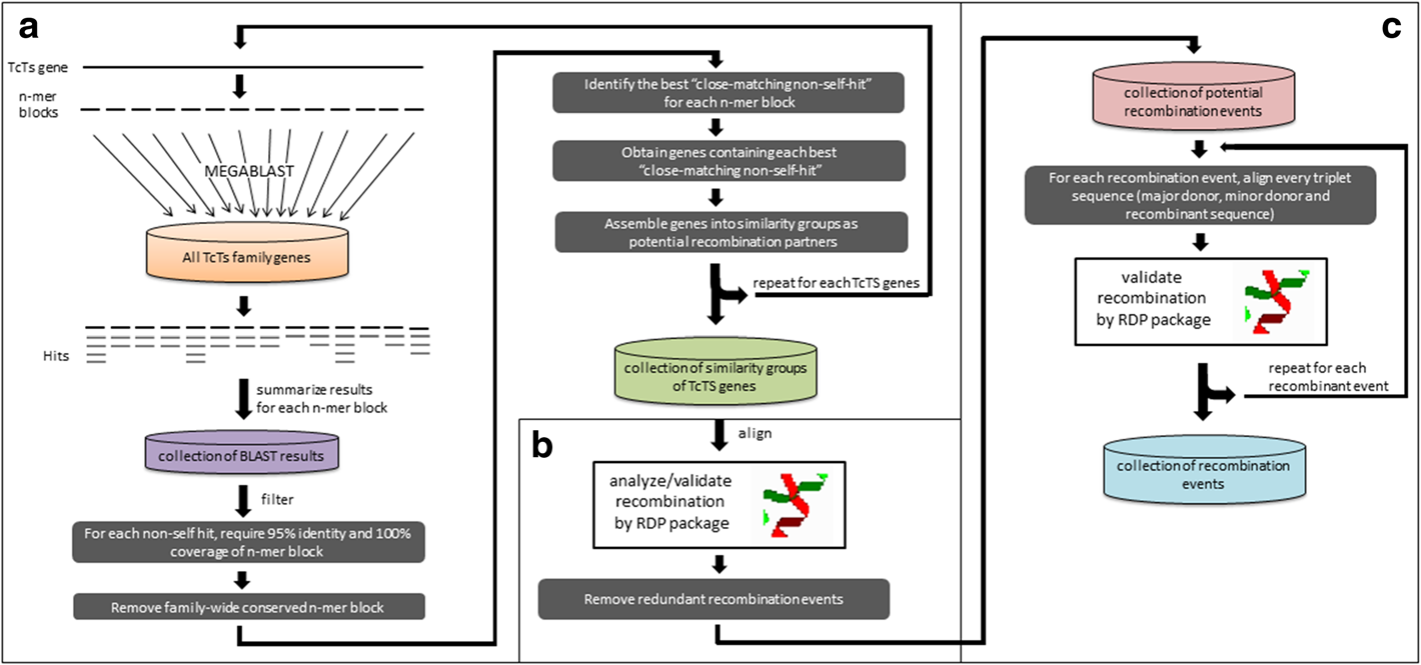
Workflow of recombination detection pipeline forTcTSgene family. **a** TcTS sequences are binned into similarity sets by scanning each TcTS gene for closest-matching non-self sequences within the TcTS gene family; **b** RDP is used to identify possible recombinant regions, recombination breakpoints, and the major and minor sequence donor(s) among the TcTS similarity sets; **c** False-positive recombination events are removed by realigning each recombination event’s triplet of mosaic sequence, major donor and minor donor

In order to more thoroughly evaluate the role of recombination in the evolution of the TcTS family, we developed a recombination detection pipeline that relied heavily on the Recombination Detection Program (RDP) v4.17, [14] (Fig. 4; see Methods). Evaluation of the pipeline with simulated data indicated dependable performance (Additional file 10: Table S4) with a sensitivity of 0.93 and 0.73 for gene-conversion-recombinant genes and gene-conversion-recombinant events, respectively, demonstrating the ability of our pipeline to accurately capture the presence of recombinant genes and recombination events. Our pipeline displayed a specificity of 0.54 and 0.44 for gene-conversion-recombinant genes and gene-conversion-recombinant events, respectively. Note that the event-specificity, which describes how frequently the pipeline precisely determines recombination breakpoints, is impacted by the difficulties of pinpointing the recombination breakpoint between sequences with less informative variations (e.g. random mutations that obscure sequence origins). The false positive rates are below 0.003 for all recombination-negative datasets tested. Notably, our recombination pipeline is not intended to detect reciprocal exchange of DNA sequences between 2 TcTS genes, as is shown by simulation results (Additional file 10: Table S4b).

**Fig. 5:**
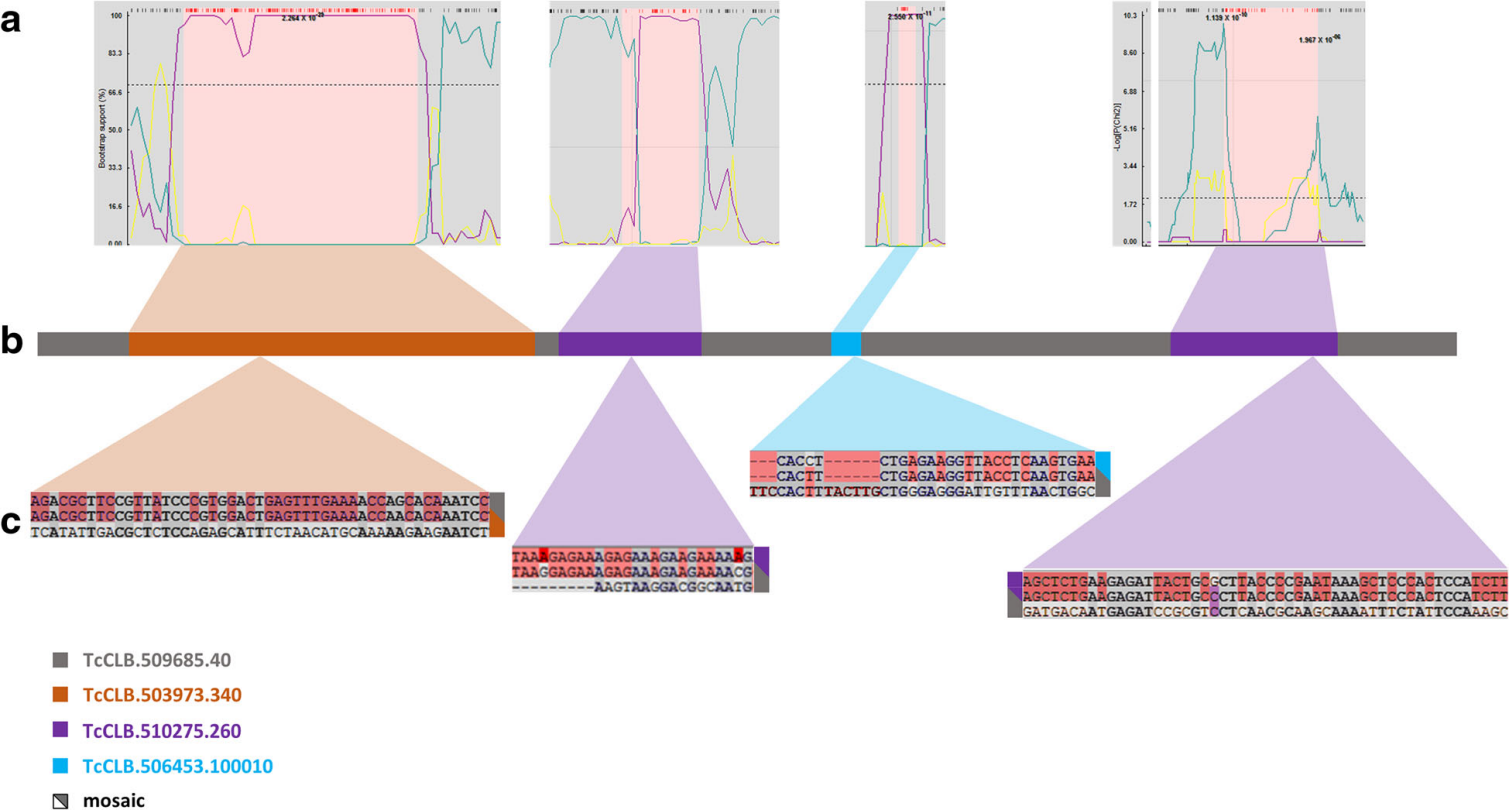
A mosaic TcTS gene showing evidence for recombination events and example sequence comparisons within each minor donor sequence. **a** BOOTSCAN and MAXICHI output showing alterations in bootstrap support and peak log(p(χ^2^)) values, respectively, at mosaic region boundaries. **b** The TcCLB.509685.40 mosaic gene with donor contributions indicated by colors. **c** Partial alignment of mosaic regions with respective donors. In each sequence triplet alignment, the middle sequence is the mosaic gene, *shading highlights* identical nucleotides of mosaic sequences with major (top) and minor (bottom) donor

**Fig. 6:**
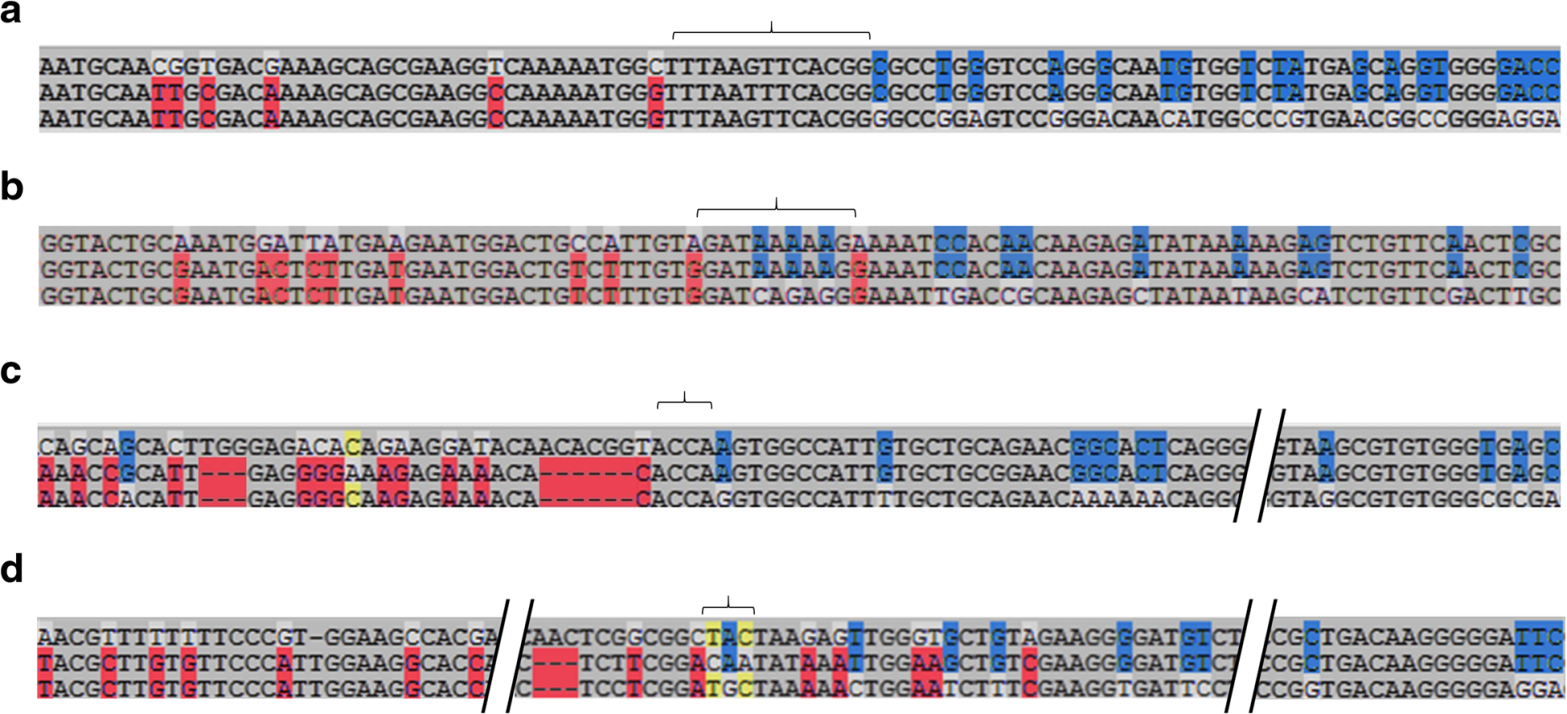
Examples of boundary regions in mosaic TcTS. From the mosaic TcCLB.509685.40 gene in Fig. 4, boundary regions on one end of each of the 4 donor sequences are shown. In each sequence triplet alignment, the middle sequence is the mosaic gene, *red* and *blue shading* highlight identical nucleotides of mosaic sequences with major donor and minor donors. The *brackets* indicate the region most likely containing the recombination breakpoint. **a** Donor sequence = TcCLB.503973.340; **b** Donor sequence = TcCLB.510275.260; **c** Donor sequence = TcCLB.506453.100010; **d** Donor sequence = TcCLB.510275.260

Using this pipeline to analyze the 3209 TcTS genes, we document clear evidence of recombination in 787 *trans*-sialidase genes with a total of 2087 recombination events; 783 and 749 genes have participated at least once as major donors and minor donors, respectively. Of all the 2087 recombination events, 1742 events have a p-value less than 0.0001 with one or more of the algorithms in RDP and 680 events have p-values <0.0001 for all algorithms (Additional file 11: Table S5). All 2087 recombination events also have multiple-test corrected p-values under 0.01. Thus, these recombination events are detected with very high confidence. The 787 mosaic genes identified have between 1 and 12 recombination events each, with an average of 2.65 recombination events/genes (Additional file 12: Figure S7). The signals of mosaic sequence patches in TcTS genes can be confirmed by manual inspection at the nucleotide level and Fig. 5 shows an example. Note that the break-points of recombinant regions frequently lack a distinct boundary but instead have short regions that are not encoded by either the major donor or minor donor (Fig. 6).

**Fig. 7:**
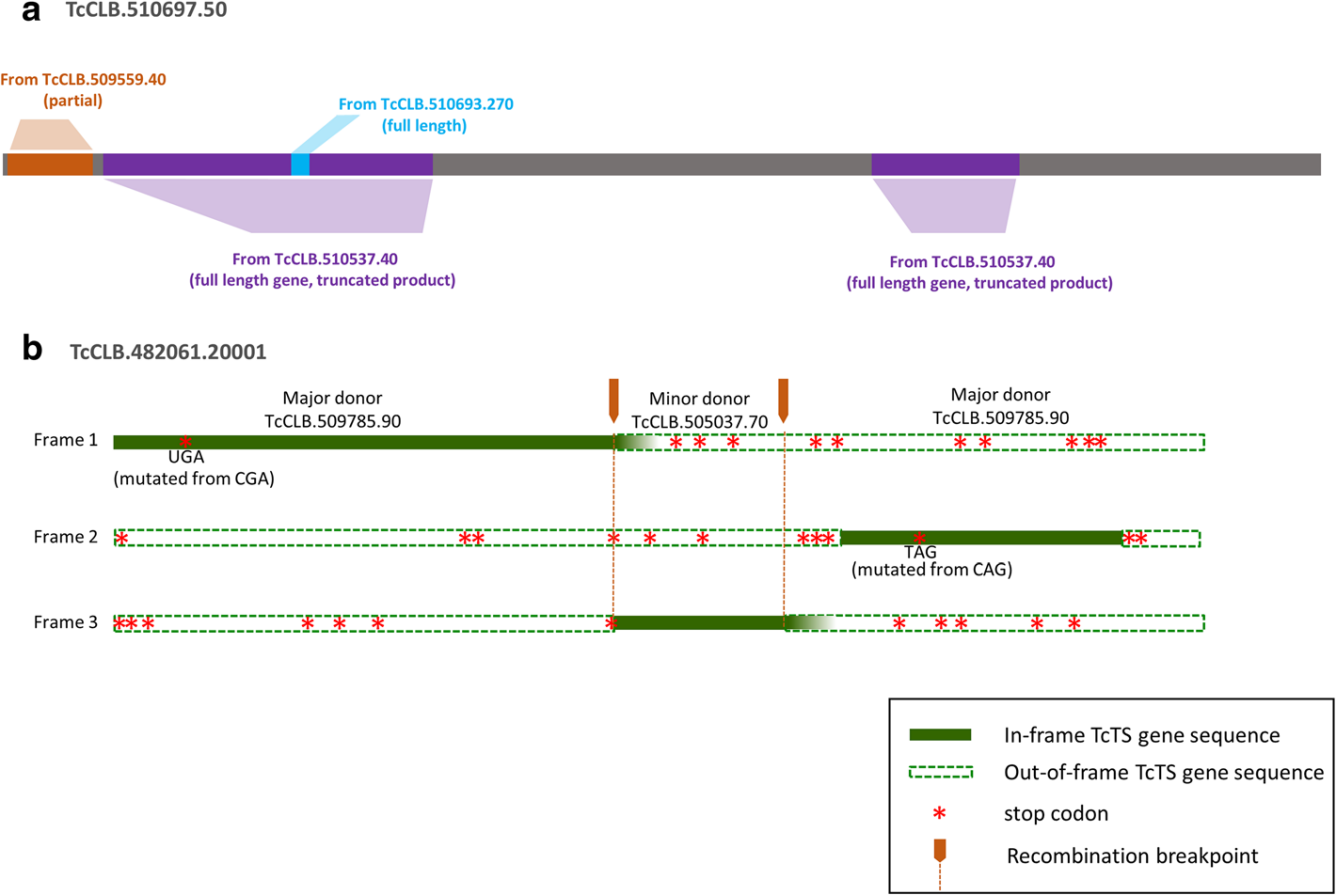
Productive and nonproductive recombination events. **a** The full length, protein coding TcTS TcCLB.510697.50 has incorporated mosaic parts from partial TcTS gene and full length TcTS donors. **b**. Introduction of sequence from the full-length TcCLB.505037.70 gene into the wrong reading frame of the major donor TcCLB.509785.90 results in a partial TcCLB.482061.20001 product. Also note that two in-frame stop codons have resulted from single point mutations in the major donor supplied sequence

Participation in recombination (either as the mosaic product or as a major or minor donor) was higher for previously annotated TcTS genes relative to the newly annotated genes (Additional file 11: Table S5, tab “summary”), although a significant proportion of this difference was due to the smaller average size of the newly annotated TcTS genes. Recombination events were more likely to be detected between full length and other TcTS genes of 2500 bases or more (Additional file 13: Figure S8). However, 25 % of the donors contributing to mosaic full length TcTS genes were of <2500 bases, demonstrating that the incomplete TcTS gene fragments in the *T. cruzi* genome play an active role in the diversity of the expressed, full length genes. Within a select set of TcTS genes grouped into eight subsets based upon multiple component analysis [15], recombination was exactly four times more likely to occur between members of the same group (444 events) than between groups (111 events; Additional file 11: Table S5). It is also notable that recombination events both contributed to the production of full length TcTS proteins from partial gene donors (Fig. 7a) as well as partial genes from full length genes due to shifts in reading frame as a result of recombination (Fig. 7b). Thus there is an active exchange between the full-length TcTS genes encoding functional proteins and partial TcTS genes and pseudogenes dispersed throughout the *T. cruzi* genome.

## Discussion

In the evolutionary arms race between the host immune system and pathogens, mammals have incorporated immense complexity into the immune system. The multi-gene segmental organization of immunoglobulin and T cell receptor genes, combined with recombination, can produce many millions of distinct antigen receptors capable of detecting and neutralizing a diverse array of pathogens. Pathogens, including eukaryotic parasites, have also evolved mechanisms to incorporate diversity into their structures in order to avoid immune destruction. The archetypal example of antigenic variation is the variant surface glycoproteins (VSG) gene family of *T. brucei,* which has more than 2000 members. Although its expression is mono-allelic, the relatively frequent switch of the expressed VSG gene enables *T. brucei* to maintain its persistence despite the constant surveillance by host immune responses. The creation of mosaic VSGs by recombination among VSG family members [16, 17] is also crucial for parasite persistence, as host immunity to previously expressed VSGs [18] accumulates over the time of infection. Likewise, *Plasmodium sp.* have evolved smaller multi-gene families encoding cell surface proteins that depend on a high frequency of recombination to produce new immune-evading variants [19, 20].

*T. cruzi* encodes several large, highly variant gene families, including the largest known family of variant surface protein-coding genes, the *trans*-sialidase family. The mostly glycosylphosphatidylinositol- (GPI)-anchored TcTS surface proteins have several essential functions in the parasite. The presumed founding members of this family provide *T. cruzi* the ability to acquire sialic acid from host donor molecules and move it to terminal glycans in a sialyl-transferase reaction. This process is apparently necessitated by the fact that *T. cruzi* lacks the ability to produce its own sialic acid, and in the absence of sialic acid, *T. cruzi* trypomastigotes are highly susceptible to host complement activation and lysis and are also poorly invasive. However, only a small proportion of the immense ts gene family, perhaps as few as 10–15 genes, actually encode enzymatically active ts. The remainder have been ascribed a number of other activities; among the more important, as lectins potentially involved in host cell attachment [21].

Despite the importance of these ts activities for the survival of *T. cruzi*, it is difficult to reconcile the huge commitment in terms of genome space that *T. cruzi* makes to the maintenance of TcTS-family genes with these noted ts functions alone. The original assembly of the reference CL Brener genome tallied nearly 1500 TcTS genes, approximately half of which were annotated as “pseudogenes”. In this study, the remapping of the original CL Brener sequence reads and re-analysis for TcTS-like sequences more than doubled the number of full and partial TcTS genes identifiable in the CL Brener genome to the current 3209 unique genes. Further, proteome analysis also confirmed the expression of at least a portion of these newly identified full length and truncated TcTS family genes. Certainly *T. cruzi* should not require >3000 full and partial length TcTS family genes to carry out the functions of sialic acid transfer and cell adhesion, and even if all the proposed functions of ts proteins are considered, performance of these activities perhaps requires a handful of genes – not >3000. This degree of gene diversity within individuals of a single species is nearly unprecedented, rivaled only by the ~2700 member VSG gene family in the related *T. brucei* [22].

A limitation of the current study is the exclusive use of the reference CL-Brener genome of *T. cruzi*. Like most reference genomes, the CL Brener reference was generated from relatively low coverage Sanger sequencing and is prone to misassembles, particularly in regions rich in multi-gene families. As additional and more deeply sequenced and better assembled genomes of *T. cruzi* become available, it will be of great interest to compare the repertoire of ts genes in independently evolving lineages in this diverse species.

The critical requirement for the cell surface expression of ts activity presents a dilemma for a pathogen like *T. cruzi.* On the one hand, lack of ts activity significantly reduces the ability of *T. cruzi* to survive in mammalian hosts. On the other, expression of a single species of ts protein on the parasite surface would provide a tantalizing target for the host immune system and an effective response to such a target could prove deadly for *T. cruzi*. Indeed ts proteins are significant targets of anti-*T. cruzi* immune responses [23] and vaccination against ts proteins is somewhat effective, at least in preventing potentially lethal infections (reviewed in [24]). Thus there is strong pressure from host immune responses on ts proteins, and as with other cases of antigen diversification in pathogens, this is the likely driving force for the retention and diversification that ultimately created this immense family of genes.

The mechanism of the expansion and continued diversification of the TcTS family in *T. cruzi* is gene duplication, mutation and recombination among family members. We present evidence for 787 mosaic *trans*-sialidase genes resulting from 2087 separate recombination events. Due to the conservative calling of recombination by gene conversion, ours is a minimal estimate; other (conversion) recombination events are likely obscured by sequence divergence resulting from accumulating mutations in both donor and recipient genes. Despite the frequency and wide scope of recombinations, all the TcTS family members, including enzymatically active TcTS, ts-inactive, truncated molecules, and gene fragments incapable of producing a protein product, retain a similar domain structure. The retention of the common features of TcTS genes and proteins across the entire family likely reflects constraints imposed by the non-enzymatic functions of TcTS, such as binding to (host) sialyl and beta-galactopyranosyl residues [25, 26], as well as the contribution of pseudogenes/partial genes to the formation of new mosaic TcTS genes. Substantial homology is generally required for gene conversion-based recombination [27, 28] and this requirement would work to sustain the common structure of the TcTS genes. The fact that intra-gene-family recombination events among TcTS genes are four times more likely to occur between genes in the same similarity group [15] than between members of different groups supports this point. Importantly our analysis additionally supplies evidence that the structurally conserved but sequence variant non-coding gene fragments are derived from and are recycled back into functional (i.e., full length, expressed) TcTS genes. Indeed, nearly half of the 437 full-length protein-coding TcTS identified as mosaics have incorporated TcTS pseudogenes and/or partial genes. Thus, the non-coding TcTS fragments undoubtedly acts as a repository of diversity for the generation of new and antigenically diverse TcTS genes.

Gene conversion has been shown to be a major intra-VSG-family recombination mechanism [16], driving antigenic diversity in *T. brucei* [29, 30]. Thus it was reasonable to suspect that gene conversion recombination events was integral to the generation of variation in the *T. cruzi* ts gene family. Classical recombination analysis typically relies on highly accurate sequence alignment and uses evidence of local disruption of sequence signatures or phylogenetic signals as signs of recombination. Rigorous statistical analysis can then be used to confirm the significance of the identified recombination breakpoint. For gene families such as TcTS containing thousands of members, such methods are not possible. Alternatively, BLAST algorithms can be used to find local sequence matches between different family members [31] or phylogenetic incompatibility distribution can be used as an index to estimate recombination activity [13] and such methods have been applied to document recombination events in the *T. brucei* VSG gene family. However, such alternative methods, unlike the classical recombination analysis, cannot ensure both statistical support and the search-comprehensiveness of recombination events. Here we propose an alternative recombination analysis pipeline that can be used on large and diverse genes families while also providing statistical validation of the results.

This recombination detection pipeline first uses BLAST to find local sequence matches between individual TcTS genes, then, for each local sequence match, we group involved-TcTS genes into a subset, between which recombination is likely to produce the mosaic patch (local sequence match). Then for each TcTS subset, we align the sequences and subject the alignment to a recombination detection program package (RDP) that implements many classical recombination detection algorithms. If a recombination event is cross-validated by multiple recombination detection algorithms, we perform an additional round of validation by realigning just the two recombination donors and the mosaic sequence and then subject the alignment to recombination analysis by RDP. This final validation step further removes false-positives caused by alignment artifacts. By starting from every possible mosaic-forming event in the gene family and validating through two rounds of rigorous recombination analysis, we combined both statistical support and search-comprehensiveness in our recombination detection pipeline for very-large gene families. Though search-comprehensiveness is our goal, our pipeline would still underestimate the recombination events in a large gene family since the rigorous statistical threshold would miss many weaker recombination signals; recombination events could be overlapping, masking each other and blurring the recombination breakpoint. Thus, we are likely able to see only a small part of the complex TcTS recombination landscape. The current analysis is also restricted to a single *T. cruzi* isolate, the genome reference CL Brener clone. Each *T. cruzi* clone will generate a distinct pattern of recombination and thus its own set of ts genes, resulting in incredibly diverse populations of parasites within and between hosts. Similar detailed analysis to that conducted here in other isolates is expected to confirm this species-wide diversity. Additionally, these mechanisms of gene family expansion almost certainly play a similar role in other large (mucins and mucin associated surface proteins (MASPs)) and perhaps smaller gene families in *T. cruzi*.

Although the generation and maintenance of diversity in the *T. cruzi* TcTS with that of the VSG genes of *T. brucei,* there are also significant differences. Most importantly, *T. cruzi* expresses the ts genes from multiple alleles concurrently rather than serial expression of VSG genes from single alleles as in the case of VSG genes *T. brucei.* TcTSs are broadly and abundantly expressed in both extracellular and intracellular stages of *T. cruzi* in mammalian hosts. Although it is not possible with the technology now available to determine if all of the TcTS genes are expressed concurrently (mapping short peptides or transcripts to >3000 related sequences is not trivial), it seems likely that the majority of those with intact 5′ expression and processing sites are being produced during one or more stages of the *T. cruzi* life cycle. Atwood et al. conservatively identified 223 expressed TcTS (both full and truncated genes) in a proteome study of all *T. cruzi* life-cycle stages, and 30 out of 50 top-scoring (high abundance) proteins in this analysis were TcTS family members [5]. The immune detection of epitopes encoded in different sets of TcTS molecules is also strong evidence of the expression of many, if not all competent TcTS genes during infection [23]. The immune evasion potential of a mutually exclusive pattern of expression of gene variants, as in the VSGs of African trypanosomes, is clear, as each switch to a new variant forces a restart of the immune response to the new immunodominant antigen. The benefits of the distinctive approach employed by *T. cruzi* of expressing many variants at once is less obvious and certainly more complex. Thus *T. cruzi* has evolved a unique immune evasion process and likely, a unique way of regulating the size of this and other gene families and the diversity among family members.

We favor the hypothesis that the expression of large numbers of TcTS variants serves an immune evasion function primarily for intracellular amastigote stages of *T. cruzi* and contributes to avoidance of detection by CD8^+^ T cells of parasite-infected cells. Expression of an abundance of different ts molecules in an infected host cell could flood the class I MHC presentation pathway with a huge diversity of potential epitopes, each competing for binding to and presentation by MHC. As a result, the effective density of most parasite epitopes could remain below that necessary to trigger effector T cells, particularly for host cells with more modest levels of MHC expression [32, 33]. The very high sequence similarity of TcTS proteins would also provide for a network of variant epitopes capable of acting as altered peptide ligands – partial agonists or antagonists – of T cells specific for related epitopes [23, 34] and references therein). The timing of expression of the TcTS proteins in intracellular *T. cruzi* amastigotes also impacts the presentation of ts epitopes on infected cells. We recently reported that the highly immunodominant TSKb20 TcTS epitope is not detectable on *T. cruzi* –infected host cells until >24 h after invasion of host cells. In contrast, a subdominant, non-variant epitope from the parasite flagellum is recognized within 3–6 h after infection [35]. Interestingly, boosting the immune response to flagellar rod proteins also significantly enhanced resistance to *T. cruzi* [35]. The impact of the high frequency ts epitope-specific CD8^+^ T cell response generated in C57Bl/6J mice is relatively minimal, since ablation of this response does not affect the ability of hosts to control of *T. cruzi* infection [36]. These data suggest that while the structure, complexity and expression of the ts family of genes does not *prevent* the generation of ts-specific CD8^+^ T cell responses, collectively these characteristics render the generated ts-specific response irrelevant with respect to the control of *T. cruzi* infection. In short, although unconventional, the simultaneous expression of many variant proteins appears to be a highly effective immune evasion strategy.

The creation of new mosaic genes via intra-family recombination likely contributes to the chronicity of a number of viral and bacterial infections [37], and this within-host evolution of antigens may also play a role in the ability of *T. cruzi* to persist for decades in humans and other hosts. Additionally the evolution of diversity among different lineages within the species – potentially resulting in a distinct sets of pathogen genetic types in each host – has implications beyond the individual host. Palmer and coworkers have argued that the potential for superinfection – the reinfection of an already infected host – also applies evolutionary selective force for the genetic diversification of antigen variants beyond that necessary for persistence within an individual host [38, 39]. This pressure would be particularly strong when the infection rates within a population are high, effectively making all members of the community accessible to infection (or re-infection), irrespective of current infection status. There is solid evidence for re-infection of currently and previously infected hosts by *T. cruzi* [40, 41], and the detection of hosts carrying mixed populations of genetically distinct parasite lineages suggests that such superinfections occur naturally [42–44]. Indeed superinfection with genetically distinct parasite types would facilitate genetic exchange within the species, a particularly important benefit in a species like *T. cruzi* that appears to rarely undergo full genome hybridization. The evidence of substantial genetic exchange between diverse *T. cruzi* lineages supports this possibility [45–48].

Solid proof of an immune evasion role for the TcTS gene family at the level of CD8^+^T cells awaits procedures which can drastically reduce the number of simultaneously expressed ts genes within *T. cruzi*. The CRISPR-Cas9 gene editing system may provide that opportunity. We have recently shown the ability in *T. cruzi* to substantially decrease the functional activity of proteins encoded by over 50 genes using the CRISPR-Cas9 system and just three guide RNAs [49]. The most highly conserved motifs identified in the TcTS proteins (Fig. 3) correlate with high DNA sequence homology that allows a relatively small number of gRNAs to target >1000 TcTS genes. Thus, we believe it is well within reach to ask if mutation of large numbers of TcTS genes compromises the immune evasion capabilities of *T. cruzi*.

## Conclusions

*T. cruzi* has evolved a unique immune evasion process that involves the simultaneous expression of large numbers of constantly changing surface molecules. Although the mechanisms for generating and maintaining the TcTS gene family are conventional - duplication, mutation and recombination – the size and diversity of the TcTS family is unprecedented. It is likely that other large gene families in *T. cruzi* utilize these same processes also driven by the need to evade host immune responses. Recently developed tools based on CRISPR/Cas9 should provide the means to substantially reduce the size and diversity of these gene families and determine the contribution of gene family size to parasite persistence in infected hosts.

## Methods

### Data sources

From version five of the *T. cruzi* genome [1] the following data were obtained: 32,746 contigs, as well as the coordinates and annotation data of predicted genes, from the National Center for Biotechnology Information (www.ncbi.nlm.nih.gov, GenBank Accession: AAHK00000000.1) and 1,131,562 whole genome shotgun sequence (WGS) reads from the Institute for Genome Research (TIGR, pathema.jcvi.org/tdb/e2k1/tca1/).

Signal peptide predictions were performed using SignalP [50], while GPI Anchor predictions were performed using DGPI [51]. Typical TcTS protein motifs are: shed acute-phase antigen (SAPA) repeats, FRIP motif (xRxP), the Asp block (SxDxGxTW) and VTVxNVxLRN motifs [4]. Among the amino acids implicated in the catalytic mechanism of TcTS proteins are Arg53, Asp59, Val95, Asp96, Tyr113, Tyr119, Trp120, Ala179, Glu230, Arg245, Pro283, Trp312, Arg314, Tyr342, and His342 [4, 52, 53]. Custom PERL scripts were used to identify these protein motifs and residues.

For the proteomic validation, two sequence databases were utilized: 1) the 32,746 genomic sequences and 2) the protein sequences from the 21,786 non-*trans*-sialidase genes from the annotated genome [1] combined with the 3209 *trans*-sialidase sequences from this study, which were concatenated with their sequence reversals (concatenated forward/reverse).

### Proteomic validation of expression of TcTS proteins

Identification of *trans*-sialidase proteins was performed using three proteomic datasets previously generated: 1) a whole proteomic analysis of all four life-cycle stages analyzed on Micromass Q-TOF-2 (Waters) [5], 2) an organelle enrichment analysis of trypomastigotes, epimastigotes, and amastigotes on LTQ-FT (Thermo Scientific), and 3) a secretory protein enrichment analysis of trypomastigotes and amastigotes on both LTQ-XL and LTQ-FT (Thermo Scientific). Collectively, the 1401 generated LC-MS/MS runs were searched against one of the proteomic databases described above: whole proteome against forward/reverse proteins, organelle enrichment against 6-frame translations of contigs, and secretory enrichment against forward/reverse proteins.

The proteomic datasets were validated and analyzed using the ProteoIQ software [PREMIER Biosoft, http://www.premierbiosoft.com/]. The whole proteome and secretome datasets were validated using a 1 % protein false-discovery rate (FDR) and 10 % peptide FDR [54]. The organelle enrichment proteome was validated using a 0.9 protein probability and 0.5 peptide probability.

### Motif and architecture analysis

MEME version 3.5.4 [11] was utilized to identify motifs in the *trans*-sialiadase protein sequences. The command-line parameters for execution were: mod = zoops(each sequence can contain zero or one occurrence of each motif), nmotifs = 100 (search for 100 motifs), maxsize = 100,000,000 (dataset size essentially unlimited), minw = 10 (minimum motif width is 10 residues), maxw = 100 (maximum motif width is 100 residues), evt = 0.01 (maximum e-value for motif is 0.01).

Custom PERL scripts were used to parse the MEME results and to output motif architectures for each TcTS sequence. The architecture is simply the in-order list of motifs (in 3-digit numbers from 001 to 100). A custom Java-tool, “MotifAlign”, was then used to align the architectures in order to determine a model TcTS architecture and to organize individual sequences into broader classes based on their architecture (Additional file 6: Figure S4).

### Entropy analysis

Sequences for TcTS genes whose transcripts were shown to be significantly expressed ([55] in the amastigote life-cycle stage were aligned using ClustalX [56]. Sequences were removed if >30 % of positions in the alignment were gaps. The remaining sequences were submitted to the Sequence Variability Server from Harvard University to determine the Shannon Entropy at each position [57]. Smoothed entropy values were calculated by averaging the entropy values in nine amino acid windows.

### Phylogenetic tree of related TcTS sequences

To obtain clusters of highly-similar TcTS suitable for phylogenetic tree construction, 930 TcTS sequences between 2000 and 3000 bp were MEGABLASTed [58] to each other using the non-default parameter-v 2000 –b 2000, and the 149 sequences with high scoring segment pairs (HSPs) covering more than 70 % of the query sequence length with a minimum sequence percent identity of 70 were selected. The setting of 70 % sequence identity was chosen to select TcTS seqeuence for subsequent tree inference because it strikes a balance between alignment quality (higher concensus identity, fewer gaps) and homolog numbers (149 homologs yielding high quality trees).

Protein sequences of the 149 highly-similar TcTS were aligned using MUSCLE algorithm [59] implemented in Geneious v5.6.5. Alignment of the TcTS sequences was manually edited with alignment viewer in Geneious v5.6.5, Bayesian tree inference was performed using MrBayes v3.2.1 [60] parallel version on XSEDE (Extreme Science and Engineering Discovery Environment) via CIPRES Science Gateway [61], non-default parameters are generalised time-reversible (GTR) substitution model (nst = 6), gamma rate variation with 8 discrete categories, nchains = 5, temp = 0.3 (more MCMC chains and higher heating temp are required to reach convergence), 10,000,000 MCMC generations, burn-in fraction 0.5. Phylogenetic trees were visuallized by Figtree v1.40 (http://tree.bio.ed.ac.uk/software/figtree/). Treeness was calculated by TreeStat v1.2 (http://tree.bio.ed.ac.uk/software/treestat/).

### Detection of recombination among TcTS genes

A computational pipeline automated using PERL scripts was developed for identifying recombination events in TcTS. First, all TcTS sequences were grouped into similarity sets by scanning each TcTS gene for closest-matching non-self sequences within the TcTS gene family (Fig. 3a). To identify these closest-matching non-self sequences, each *trans*-sialidase sequence (termed the “group identifier gene”) was first split into non-overlapping 50-mers and each 50-mer MEGABLASTed [58] against a database containing all TcTS sequences using default parameter settings. For each 50-mer, the closest matching TcTS gene with 100 % coverage was identified; the similarity group for each TcTS gene consisted of the closest-matching non-self-hits for all 50-mers from that gene. Conserved regions identified by having multiple identical closest-matching hits, were excluded. Trials varying the size of the split window and overlap between windows showed the 50-mer, 0 overlap setting to provide adequate sensitivity without being computationally burdensome.

To identify possible recombinant regions, recombination breakpoints, and the major and minor sequence donor(s) among the *trans*-sialidase sequence groups, each group of sequences was aligned by Clustalw2 (v2.1) [56] and each alignment then analyzed for recombination by RDP (Recombination Detection Program) v4.17, [14] using the following parameters: Linear sequence, Highest acceptable *P*-Value = 0.01, Bonferroni correction, no permutations, check alignment consistency (Fig. 3b). The settings for the individual algorithms are provided in Additional file 14: Table S6. RDP-predicted recombination events were further filtered to meet three criteria: (1) the recombination event is detected by at least two algorithms, (2) the detected mosaic sequence must be the group identifier gene, and (3) redundant recombination events, characterized by recombination events with the same recombinant region in same mosaic gene with distinct, but highly similar donors, are removed.

Since RDP relies on accurate alignment as input for analysis of recombination events, alignment artifacts introduced by distantly related sequences in each sequence group might create false recombination signals (as we observed), despite the fact that RDP checks for alignment consistency before analyzing for recombination. As a final step, the sequences predicted to contribute to each recombination event were realigned using only the predicted mosaic gene, the major donor gene and the minor donor gene for that event, and the alignment resubmitted to recombination analysis and resulting events again filtered as described above (Fig. 3c).

To evaluate the specificity and sensitivity of our of recombination detection pipeline, simulated *trans*-sialidase gene family datasets with and without recombination were generated as negative and positive datasets, respectively, and then the recombination results using our pipeline was compared to the true recombination event in the control dataset. A similarity tree was calculated for the *T.cruzi trans*-sialidase gene family using the Tamura-Nei genetic distance model and the neighbor-joining tree constructing method [62]. DAWG v1.2 [63] was then used to generate simulated *T.cruzi trans*-sialidase gene families using the similarity tree of real data, GTR evolution model [64], alpha = 0.5, root sequence length 2500. The four recombination-negative datasets were generated containing random point mutation rates of 0, 1, 5 and 10 %, respectively. Homologous recombination events or gene conversion events were separately introduced into the 0 % point mutation negative data, to generate the two recombination- positive datasets (for additional details on introducing recombination events into the dataset see Additional file 15).

After the simulated datasets were analyzed by our recombination detection pipeline, the results were compared to the logged *in silico* recombination events. Sensitivity is defined as the ratio of detected recombination events over all recombination events in the simulated dataset, and specificity was defined as the ratio of the correctly identified recombination events over all detected positives event. In negative datasets, all detected recombination genes were considered false positives. In recombination positive datasets, only the detected events that had a recombination breakpoint within 200 bp distance from the real recombination break point were considered to match (correctly identified) the documented recombination events. All matched (correctly identified) recombination events were considered true positives and those that did not match were considered false positives.

## Additional files

Additional file 1: Figure S1. Flowchart to determine new putative TcTS sequences. Step 1: The sequences from the 1430 annotated TcTS genes of the current genome plus up to 1Kb flanking sequence on both the 5′ and 3′ ends were BLASTed (BLASTN) against the 1,131,562 wgs sequence reads, resulting in over 257,824 matching reads. Step 2: resulting reads were BLASTed (BLASTN) against the contigs of the current genome to map them to the most homologous location, resulting in nearly 130k reads mapping to contigs containing no annotated genes. The regions were collapsed into 9852 distinct contig regions. Step 3: the resulting contig regions were BLASTed (BLASTX) against the 735 “full length” TcTS genes in the annotated genome to identify TcTS-like sequences, resulting in 2007 candidate TcTS sequences. Step 4: the candidate sequences were BLASTed (BLASTP) against the entire TSKTSC genome to verify that the top hit was a TcTS sequence. Discarding those that were not resulted in 1780 additional TcTS sequences. (TIF 2089 kb) Additional file 2: Table S1. Newly Annotated and Modified TcTS. **(a)** 735 TcTS genes annotated as “real”; full-length containing both signal peptide and GPI anchor motifs and whose amino acid translations resulted in no in-frame stop codons; **(b)** List of all TcTS genes; **(c)** List of newly annotated TcTS and those whose bounds were modified in this study. Temporary IDs were assigned to each sequence by adding “2000” followed by a unique identifier for each newly discovered sequence on a particular contig and by pre-pending “1000” to the unique identifier for each modified sequence. **(d)** Locations of all TcTS genes and **(e)** newly identified, and modified TcTS on the *T. cruzi* chromosomes. Nucleotide sequences are indicated as “full-length” if they encoded both a signal sequence and GPI-anchor addition site, “partial” if encoding a signal sequence but not a GPI anchor addition site, and “pseudogene” if lacking a signal sequence. Sequences whose translations contain in-frame stop codon(s) are annotated with “in-frame stop” **(f)** List of 40 “model” full-length TcTS genes of at least 2Kb in length and containing both a signal peptide and GPI anchor motif. (XLSX 572 kb) Additional file 3: Figure S2. Merging of *trans-*sialidase sequences annotated as separate genes. The region of contig “TcCLB.505267” containing both TcTS genes TcCLB.505267.20 and TcCLB.505267.10 is shown (“TcCLB.505267.10-20”) aligned with a different, previously annotated TcTS sequence (“TcCLB.506345.90”). The previously annotated region for TcCLB.505267.20 is highlighted in brown, while the previously annotated region for TcCLB.505267.10 is highlighted red. Though these sequences were previously annotated as individual genes, CLUSTAL alignment with the other, longer TcTS sequence indicates that the entire region represents a single TcTS sequence, and thus the two separate genes were merged during re-annotation to create a single gene. Note also the added, previously unannotated region on the N-terminus of the sequence (region 1–205). (TIF 3636 kb) Additional file 4: Figure S3. “Correction” of translation for TcTS sequences containing in-frame stop codons. **(a)** Translating the DNA sequences for many TcTS genes results in one or more in-frame stop codons and out-of-frame translations (yellow bar w/ asterisks). The red bar depicts a “real” TcTS protein with no frame shifts. The pink shaded regions depict sequence homology between the two sequences when the reading frame is correct for the top protein. **(b)** When the reading frames were adjusted to maintain a TcTS-like protein sequence by removing one or more amino acids to maintain homology, the “corrected” sequence for the top protein shows high homology to other TcTS proteins throughout the coding sequence (entire region is pink). Note that we place an “X” in the protein sequence at the location of the frame-shifts. **(c)** The corrected sequence can then be utilized for protein analyses. In the example, we show motifs identified throughout the sequence, thus providing a sequence architecture. We also show the identification of a peptide that is unique to this sequence (e.g. TcCLB.509539.10) that was identified via proteomic analysis. (TIF 722 kb) Additional file 5: Table S2. Proteomic Confirmation of TcTS. **(a)** Summary of proteomic analysis. The “Min” value represents the minimum number of proteins (and their peptides) that could account for all identified peptides (see “TOP PROTEIN” in b and c), while the “Max” value is the total number of proteins (and their peptides) that contain the identified peptides (e.g. isoforms and related proteins). **(b)** List of all identified TcTS proteins. **(c)** List of identified newly identified TcTS proteins. (XLSX 64 kb) Additional file 6: Figure S4. Alignment of TcTS Architectures. Of the 3209 TcTS sequences in this study, 3176 contained at least 1 motif with an e-value of 1e-01 as determined by the described MEME analysis. The architectures, the in-order sequence of motifs, of each TcTS sequence were extracted and aligned. The alignment was constructed using the following steps: 1) identify a set of “anchor” motifs (high-confidence motifs that are present in more than 1000 sequences); 2) determine the most common order of these anchor motifs across all architectures; 3) create a preliminary model using only the anchor motifs (gaps created between non-contiguous anchor motifs); 4) find the best fit of remaining motifs to the preliminary model; and 5) determine the final model based on the motif occurrences at each position (i.e. the motif that matched to the highest number of TcTS sequences). (TIF 742 kb) Additional file 7: Table S3. Alignment and Analysis of TcTS Architectures. **(a)** Of the 3209 TcTS sequences in this study, 3176 contained at least 1 motif with an e-value of 1e-01 as determined by the described MEME analysis. The architectures, the in-order sequence of motifs, of each TcTS sequence were extracted and aligned as described. (**b**) Confidence heat map. Top row contains the motif numbers of the “model” TcTS architecture, where the model is determined by choosing the motif that is most frequent over all TcTS at each position. The cells are colored red if the model motif at that position is found in >1000 sequences, black for motifs preceding the signal peptide sequence or in the hyper-variable region containing SAPA repeats, and blue otherwise. The 2^nd^ row displays a bar-graph of the minimum smoothed entropy for the motif region. The remainder of the table is color-coded according to the “confidence score” of the motif-to-architecture for each cell. The confidence score is calculated as: (-1 * log_e_(MEME e-value))^1.5^ and the colors range from white (low) to blue (mid) to red (high). Note that the score reflects the assigned motif at each position regardless of whether it matches the model. (XLSX 1439 kb) Additional file 8: Figure S5. Architecture of Model TcTS and Mapped to 1S0I_A Crystal Structure. The model TcTS architecture (starting with the signal peptide motifs) is depicted. Each box represents an individual motif from the MEME analysis and is based on the alignment of motifs in Additional file 4: Table S4a. The top number is the motif number, the middle percentage is the representation of that motif across all TcTS sequences (including where absent), and the bottom number is the representation of that motif in TcTS sequences of length >2000b. Motifs not mapped to crystal structure are color-coded white (either not part of crystal, like the signal peptide, or the sequence homology was not high enough to constitute a match). Otherwise, frequent motifs (>1000 occurrences in all TcTS sequences) are color-coded red, while less frequent motifs are blue. The highly-variable C-terminal region containing multiple motifs is represented as a black box. Note that motif 44 (in yellow) is homologous to 1S0I_A and is shown; in the model TcTS architecture this position is occupied by motif 26. Locations of previously characterized TcTS motifs and residues are also shown. (TIF 970 kb) Additional file 9: Figure S6. TcTS Architecture Heat Map. Top row contains the motif numbers of the “model” TcTS architecture, where the model is determined by choosing the motif that is most frequent over all TcTS at each position from the aligned architectures in Additional file 4: Table S4a. The cells are colored red if the model motif at that position is found in >1000 sequences, black for motifs preceding the signal peptide sequence or in the hyper-variable region containing SAPA repeats, and blue otherwise. The 2^nd^ row displays a bar-graph of the *minimum* smoothed entropy for the motif. The remainder of the table is color-coded according to the “confidence score” of the motif-to-architecture for each cell. The confidence score is calculated as: (−1 * log_e_(MEME e-value))^1.5^ and the colors range from white (low) to blue (mid) to red (high). Note that the score reflects the assigned motif at each position regardless of whether it matches the model. (TIF 1512 kb) Additional file 10: Table S4. Summary of evaluation of the recombination detection pipeline with simulated data. a) false positive rates, sensitivity, and b) **s**pecificity of the recombination detection pipeline using simulated datasets of *trans*-sialidase gene family members, recombination positive data contains 200 recombinant genes with 1000 recombinant events. (DOCX 68 kb) Additional file 11: Table S5. Recombination events. **a)** Detailed information of 2087 recombination events detected in TcTS gene family. **b)** Summary of number and percentage of newly annotated TcTS and previously annotated TcTS participating in recombination events as recombinant product, major donor or minor donor. (XLSX 549 kb) Additional file 12: Figure S7. Recombination frequency distribution. The distribution of recombination frequency summarized for each of all TcTs gene analyzed in this study. (TIF 521 kb) Additional file 13: Figure S8. TcTS genes participating in recombination in relation to gene size. The fraction of TcTS genes in each size class that served as a minor donor, major donor or are the mosiac product of recombination. Percent active as mosaic gene was calculated by dividing the number of mosaic gene by the total number of TcTS genes for each category; Percent active as major donor was calculated by dividing the number of genes that took role (at least once) as major donor by the total number of TcTS genes for each category; Percent active as minor donor was calculated by dividing the number of genes that took role (at least once) as minor donor by the total number of TcTS genes for each category. (TIF 565 kb) Additional file 14: Table S6. Algorithms and respective parameters for the RDP package. (DOCX 17 kb) Additional file 15: Generation of control recombination positive dataset. Two types of recombination events were introduced to simulated sequences: gene conversion and homologous recombination (reciprocal exchange). For gene conversion recombination, a subsequence from a randomly chosen donor gene is extracted, and used to replace the homologous subsequence in a randomly chosen recipient gene. For homologous recombination (reciprocal exchange), homologous subsequences from two randomly chosen genes are extracted and exchanged. Normal distribution is used to model the length of the recombination region. Exponential distribution is used to model the number of recombination events per gene. Exponential distribution with probability density function *y* = 0.52622*e* ^− 0.52622*x*^ is a good approximation of the distribution of frequency of number of recombination events per gene observed in our preliminary analysis. A custom perl program script was used to introduce multiple recombination events in a single dataset. The perl script takes 4 parameters, percentage of Gene conversion recombination“g%”, total recombination gene number “n”, recombination region mean length, “l” and standard deviation of recombination region length, “std”. The program randomly chose g% of the genes to introduce recombination into. For each recombinant gene, the program sampled the exponential distribution described above to determine the number of recombination events to introduce into the gene. For each recombination event, g% probability to introduce gene conversion event, and 1-g% probability to introduce homologous recombination events (reciprocal exchange), the length of recombination event was sampled from the normal distribution (l, std^2^). (ZIP 23 kb) Additional file 16: Motif and MEME scripts. (RAR 42 kb)
