# Supplementary figures and images for: Recombination-driven generation of the largest pathogen repository of antigen variants in the protozoan Trypanosoma cruzi
Source: BMC Genomics. 2016 Sep 13;17(1):729. doi: 10.1186/s12864-016-3037-z (PMC5020489; doi:10.1186/s12864-016-3037-z)

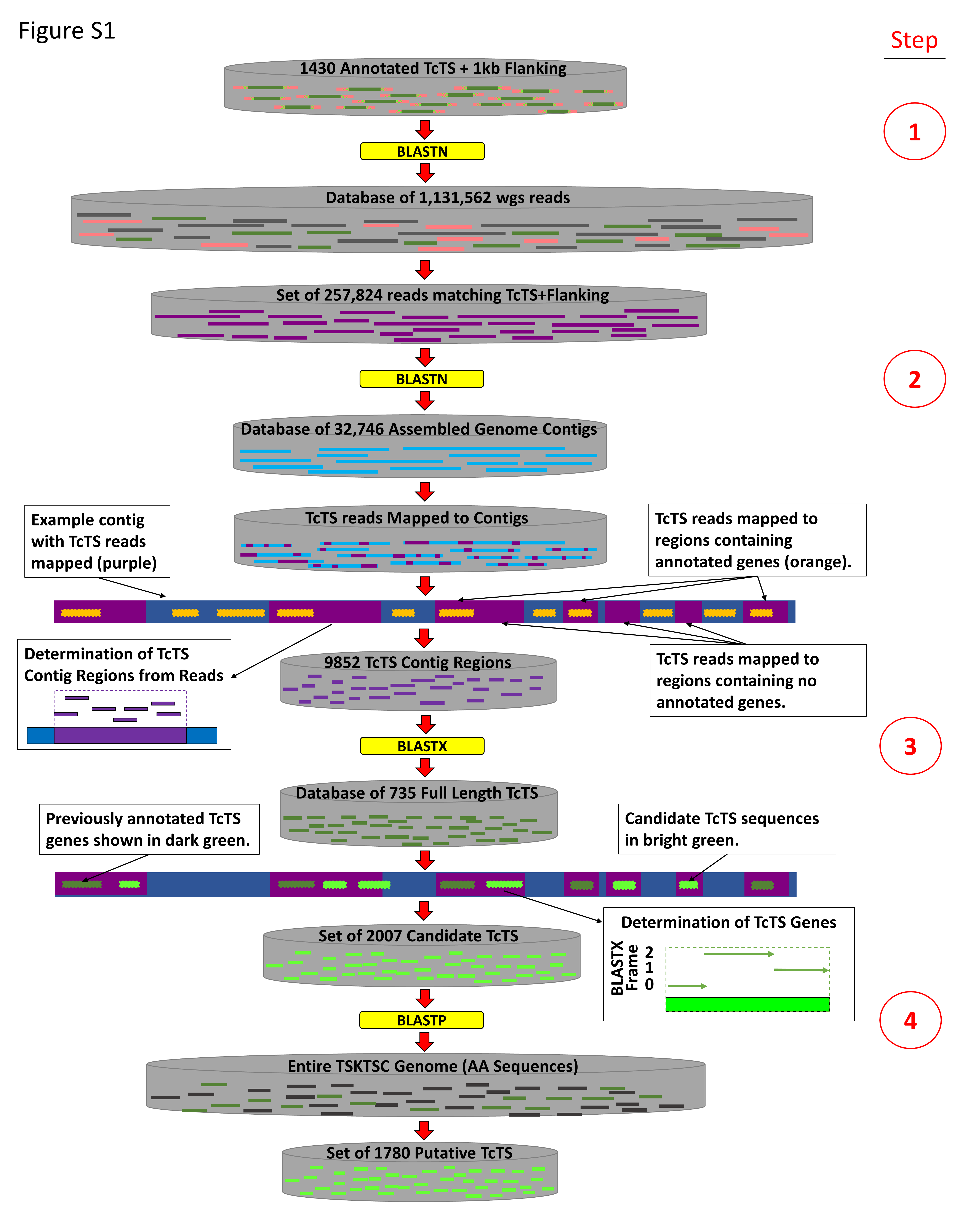

Supplement: Additional file 1: Figure S1. — Flowchart to determine new putative TcTS sequences. Step 1: The sequences from the 1430 annotated TcTS genes of the current genome plus up to 1Kb flanking sequence on both the 5′ and 3′ ends were BLASTed (BLASTN) against the 1,131,562 wgs sequence reads, resulting in over 257,824 matching reads. Step 2: resulting reads were BLASTed (BLASTN) against the contigs of the current genome to map them to the most homologous location, resulting in nearly 130k reads mapping to contigs containing no annotated genes. The regions were collapsed into 9852 distinct contig regions. Step 3: the resulting contig regions were BLASTed (BLASTX) against the 735 “full length” TcTS genes in the annotated genome to identify TcTS-like sequences, resulting in 2007 candidate TcTS sequences. Step 4: the candidate sequences were BLASTed (BLASTP) against the entire TSKTSC genome to verify that the top hit was a TcTS sequence. Discarding those that were not resulted in 1780 additional TcTS sequences. (TIF 2089 kb) [file 12864_2016_3037_MOESM1_ESM.tif]

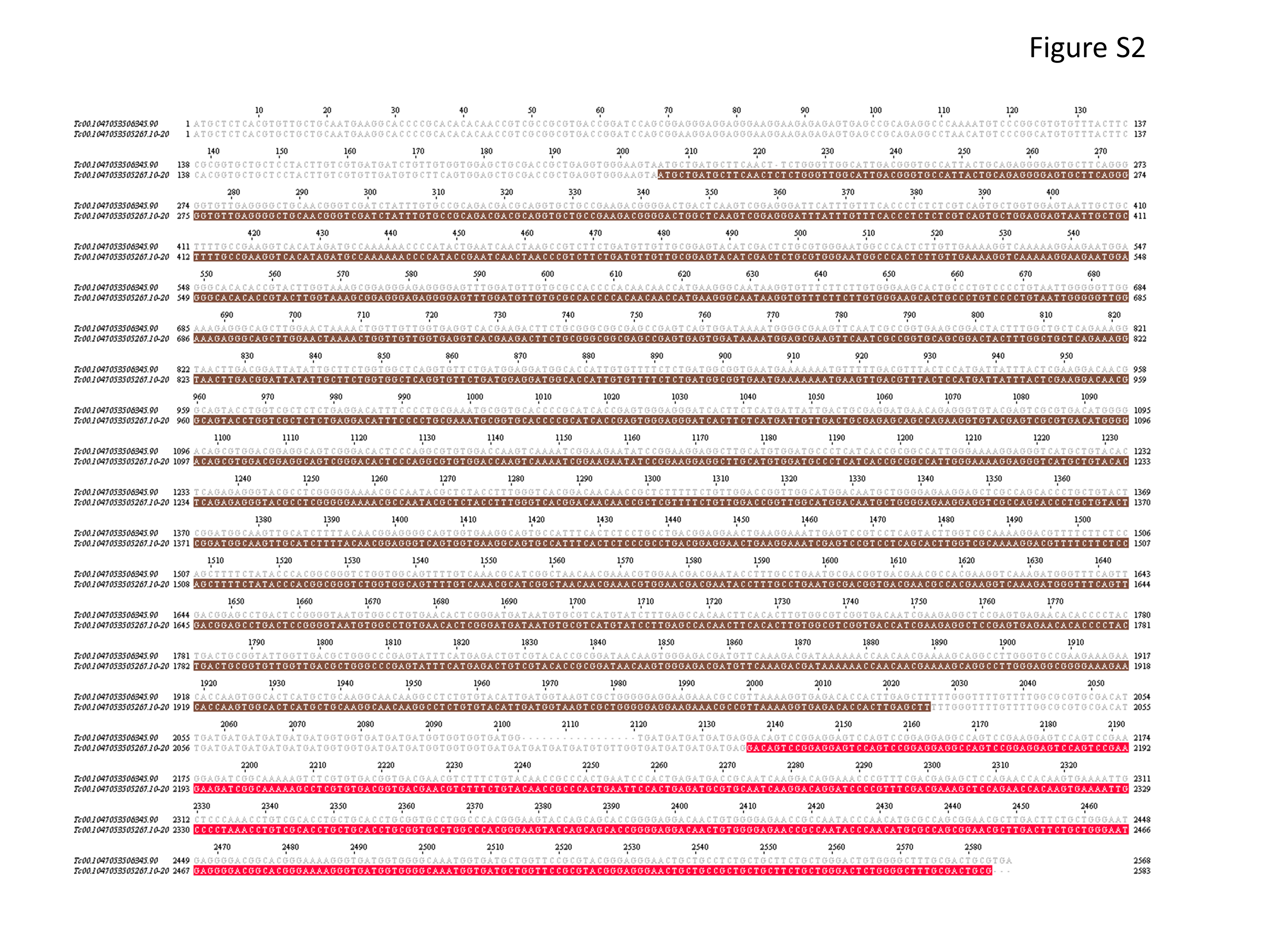

Supplement: Additional file 3: Figure S2. — Merging of trans-sialidase sequences annotated as separate genes. The region of contig “TcCLB.505267” containing both TcTS genes TcCLB.505267.20 and TcCLB.505267.10 is shown (“TcCLB.505267.10-20”) aligned with a different, previously annotated TcTS sequence (“TcCLB.506345.90”). The previously annotated region for TcCLB.505267.20 is highlighted in brown, while the previously annotated region for TcCLB.505267.10 is highlighted red. Though these sequences were previously annotated as individual genes, CLUSTAL alignment with the other, longer TcTS sequence indicates that the entire region represents a single TcTS sequence, and thus the two separate genes were merged during re-annotation to create a single gene. Note also the added, previously unannotated region on the N-terminus of the sequence (region 1–205). (TIF 3636 kb) [file 12864_2016_3037_MOESM3_ESM.tif]

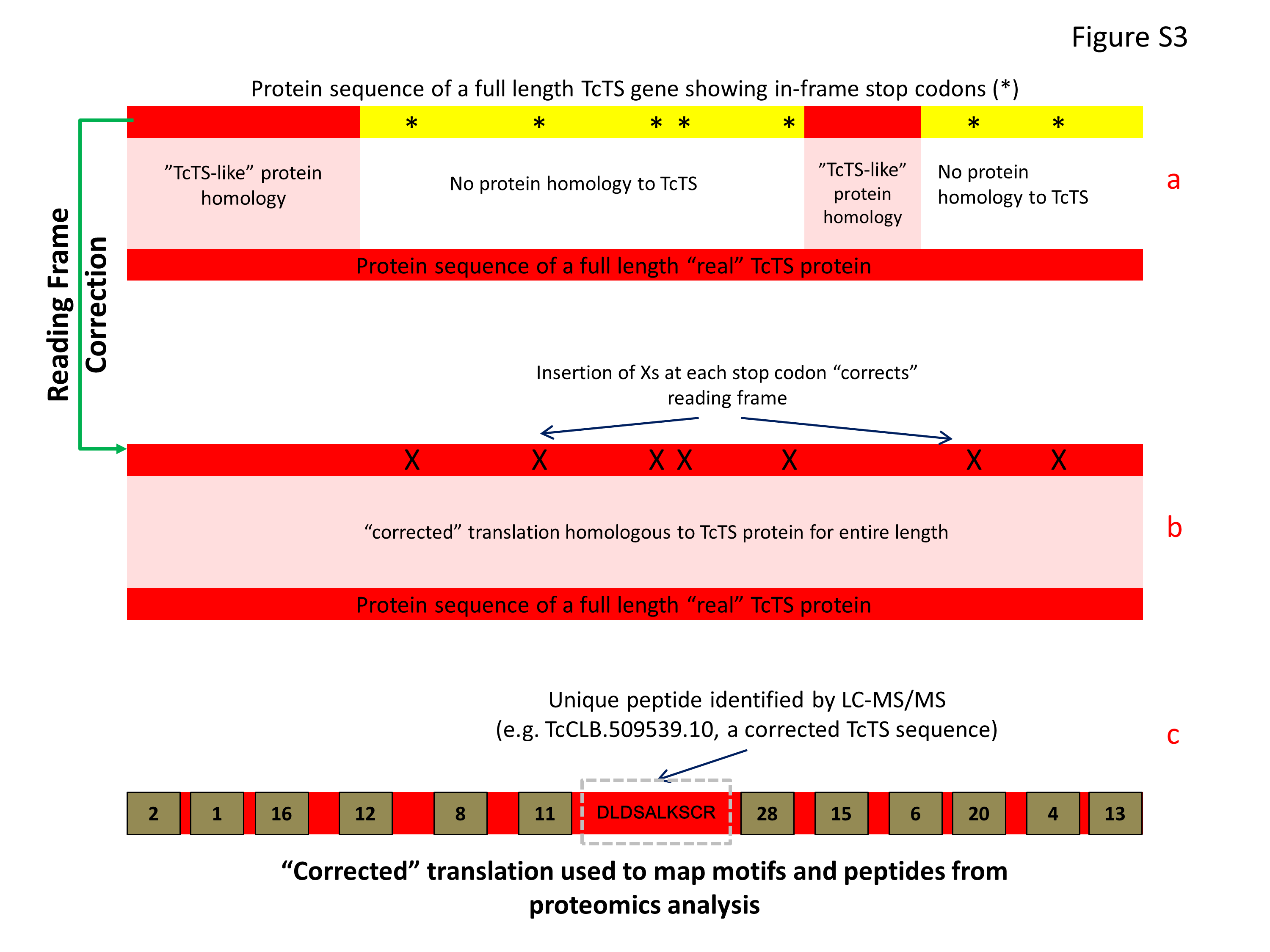

Supplement: Additional file 4: Figure S3. — “Correction” of translation for TcTS sequences containing in-frame stop codons. (a) Translating the DNA sequences for many TcTS genes results in one or more in-frame stop codons and out-of-frame translations (yellow bar w/ asterisks). The red bar depicts a “real” TcTS protein with no frame shifts. The pink shaded regions depict sequence homology between the two sequences when the reading frame is correct for the top protein. (b) When the reading frames were adjusted to maintain a TcTS-like protein sequence by removing one or more amino acids to maintain homology, the “corrected” sequence for the top protein shows high homology to other TcTS proteins throughout the coding sequence (entire region is pink). Note that we place an “X” in the protein sequence at the location of the frame-shifts. (c) The corrected sequence can then be utilized for protein analyses. In the example, we show motifs identified throughout the sequence, thus providing a sequence architecture. We also show the identification of a peptide that is unique to this sequence (e.g. TcCLB.509539.10) that was identified via proteomic analysis. (TIF 722 kb) [file 12864_2016_3037_MOESM4_ESM.tif]

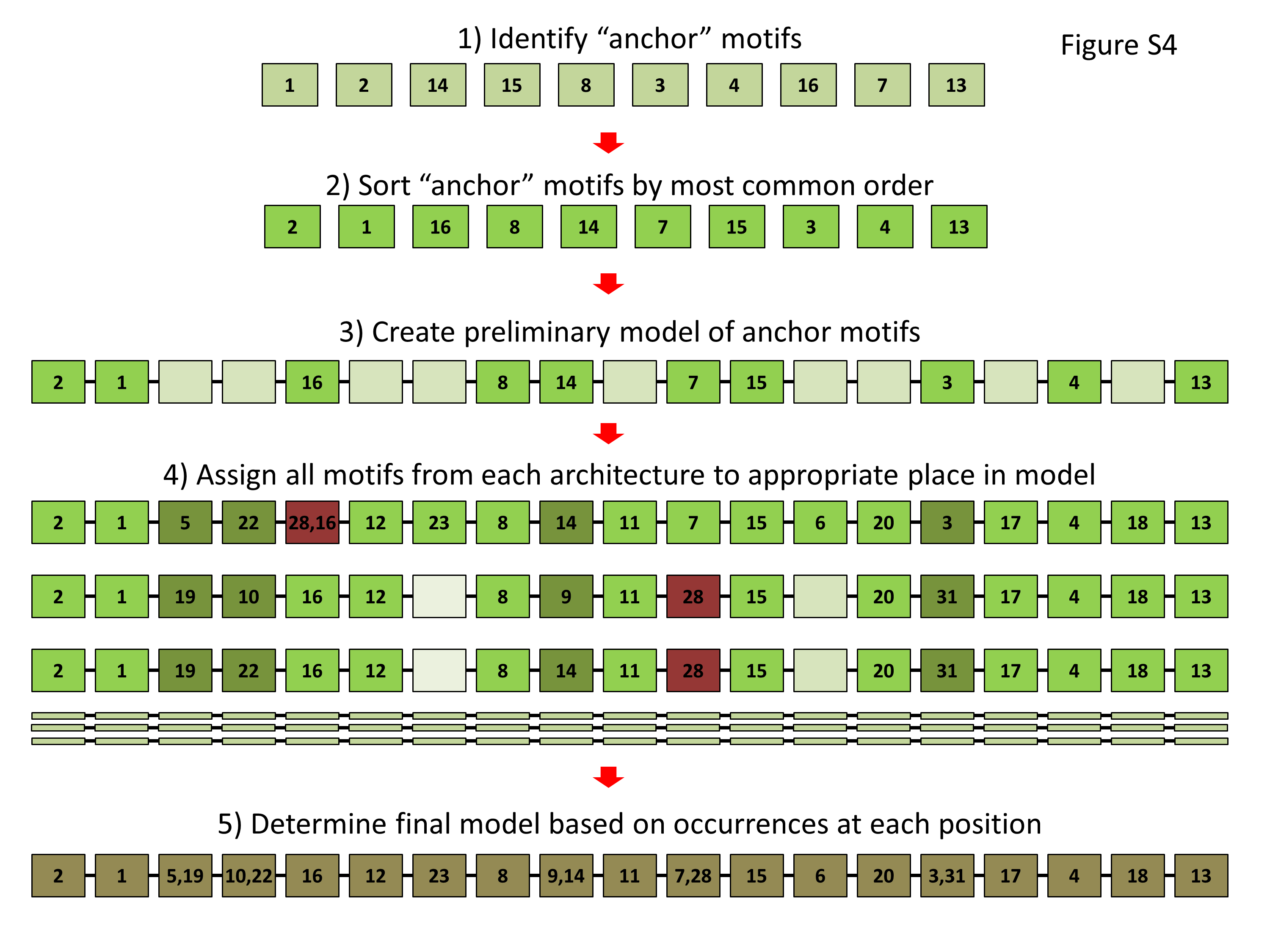

Supplement: Additional file 6: Figure S4. — Alignment of TcTS Architectures. Of the 3209 TcTS sequences in this study, 3176 contained at least 1 motif with an e-value of 1e-01 as determined by the described MEME analysis. The architectures, the in-order sequence of motifs, of each TcTS sequence were extracted and aligned. The alignment was constructed using the following steps: 1) identify a set of “anchor” motifs (high-confidence motifs that are present in more than 1000 sequences); 2) determine the most common order of these anchor motifs across all architectures; 3) create a preliminary model using only the anchor motifs (gaps created between non-contiguous anchor motifs); 4) find the best fit of remaining motifs to the preliminary model; and 5) determine the final model based on the motif occurrences at each position (i.e. the motif that matched to the highest number of TcTS sequences). (TIF 742 kb) [file 12864_2016_3037_MOESM6_ESM.tif]

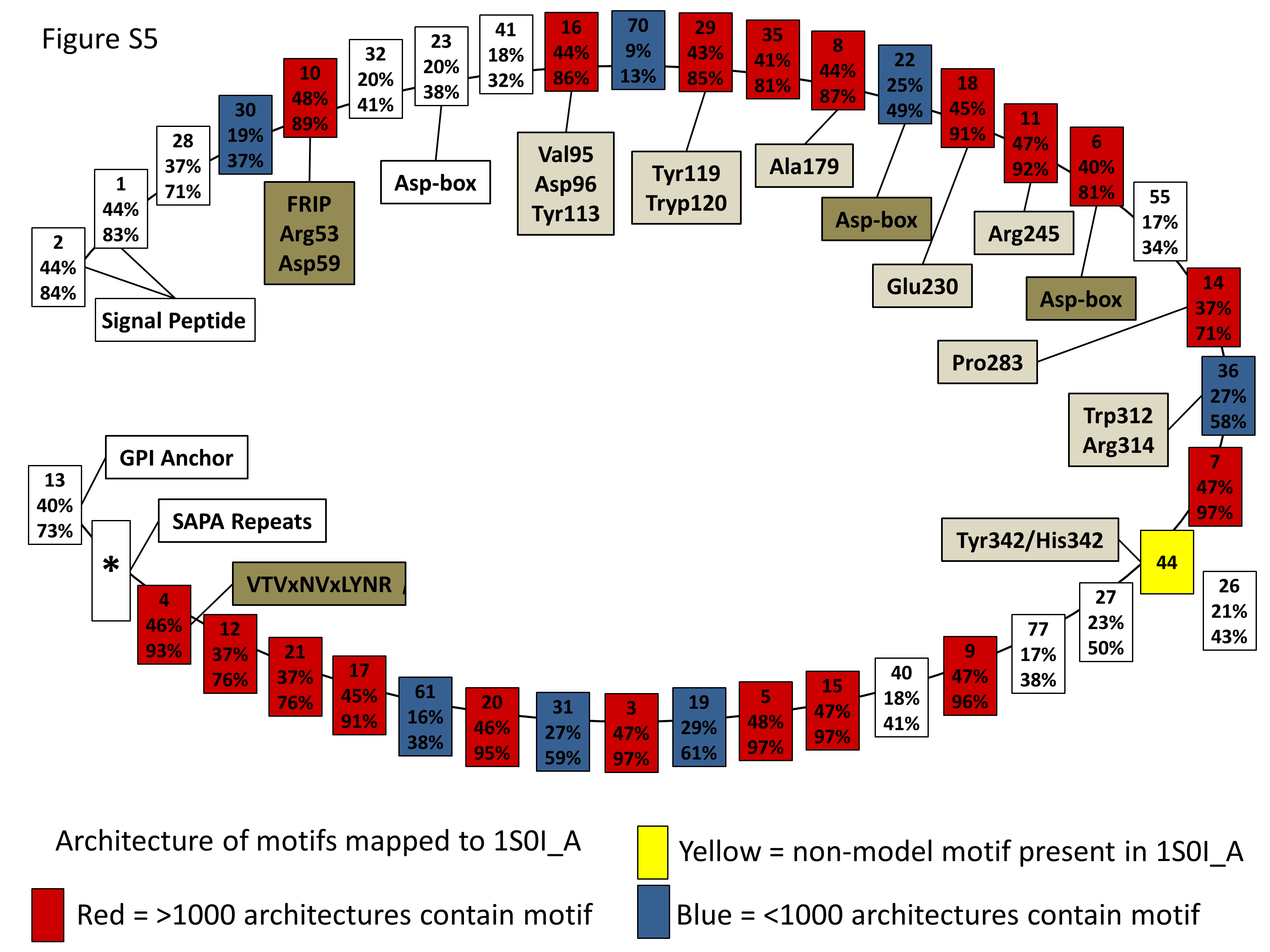

Supplement: Additional file 8: Figure S5. — Architecture of Model TcTS and Mapped to 1S0I_A Crystal Structure. The model TcTS architecture (starting with the signal peptide motifs) is depicted. Each box represents an individual motif from the MEME analysis and is based on the alignment of motifs in Additional file 4: Table S4a. The top number is the motif number, the middle percentage is the representation of that motif across all TcTS sequences (including where absent), and the bottom number is the representation of that motif in TcTS sequences of length >2000b. Motifs not mapped to crystal structure are color-coded white (either not part of crystal, like the signal peptide, or the sequence homology was not high enough to constitute a match). Otherwise, frequent motifs (>1000 occurrences in all TcTS sequences) are color-coded red, while less frequent motifs are blue. The highly-variable C-terminal region containing multiple motifs is represented as a black box. Note that motif 44 (in yellow) is homologous to 1S0I_A and is shown; in the model TcTS architecture this position is occupied by motif 26. Locations of previously characterized TcTS motifs and residues are also shown. (TIF 970 kb) [file 12864_2016_3037_MOESM8_ESM.tif]

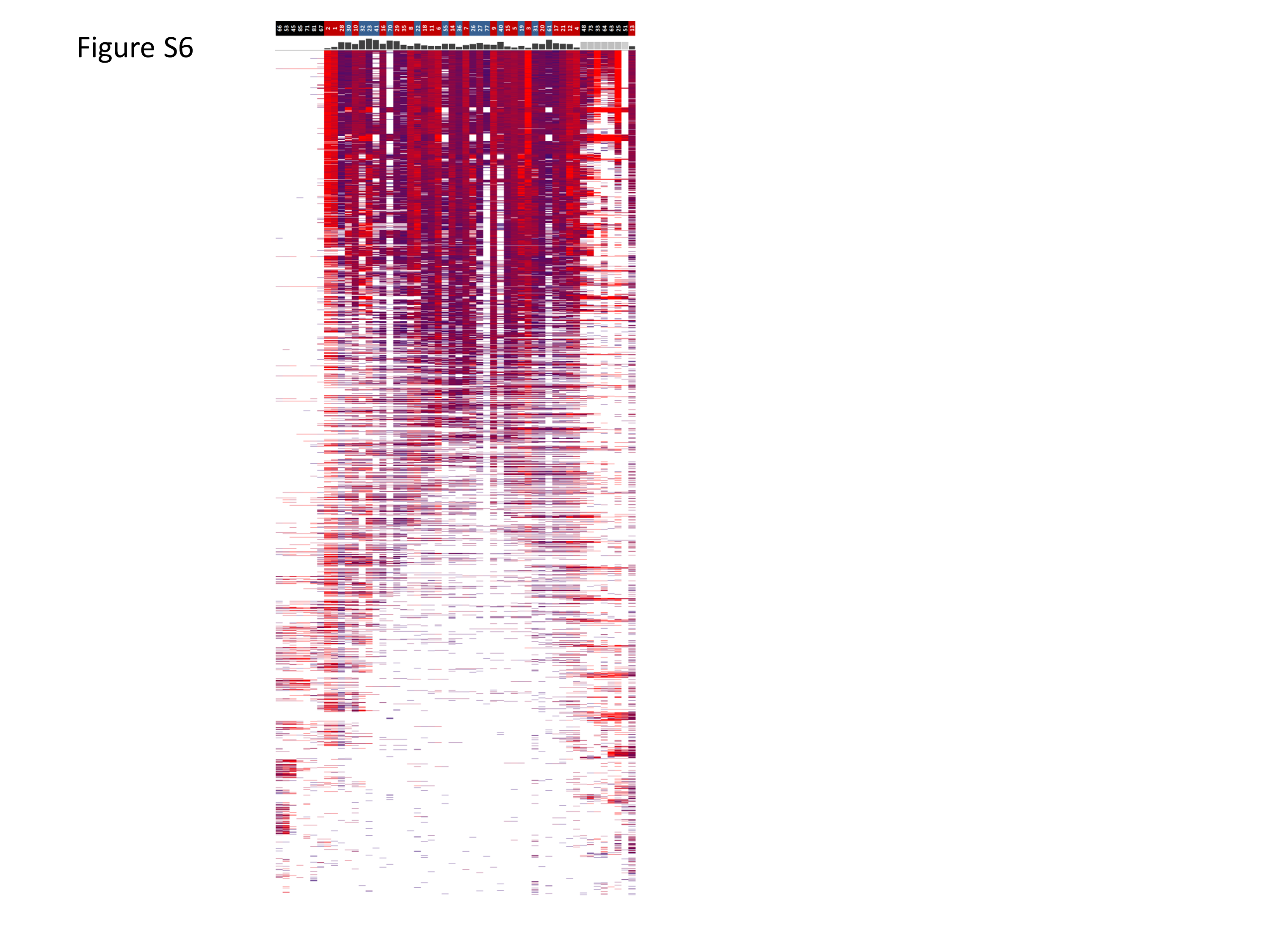

Supplement: Additional file 9: Figure S6. — TcTS Architecture Heat Map. Top row contains the motif numbers of the “model” TcTS architecture, where the model is determined by choosing the motif that is most frequent over all TcTS at each position from the aligned architectures in Additional file 4: Table S4a. The cells are colored red if the model motif at that position is found in >1000 sequences, black for motifs preceding the signal peptide sequence or in the hyper-variable region containing SAPA repeats, and blue otherwise. The 2nd row displays a bar-graph of the minimum smoothed entropy for the motif. The remainder of the table is color-coded according to the “confidence score” of the motif-to-architecture for each cell. The confidence score is calculated as: (−1 * loge(MEME e-value))1.5 and the colors range from white (low) to blue (mid) to red (high). Note that the score reflects the assigned motif at each position regardless of whether it matches the model. (TIF 1512 kb) [file 12864_2016_3037_MOESM9_ESM.tif]

**Table S4**

**a**


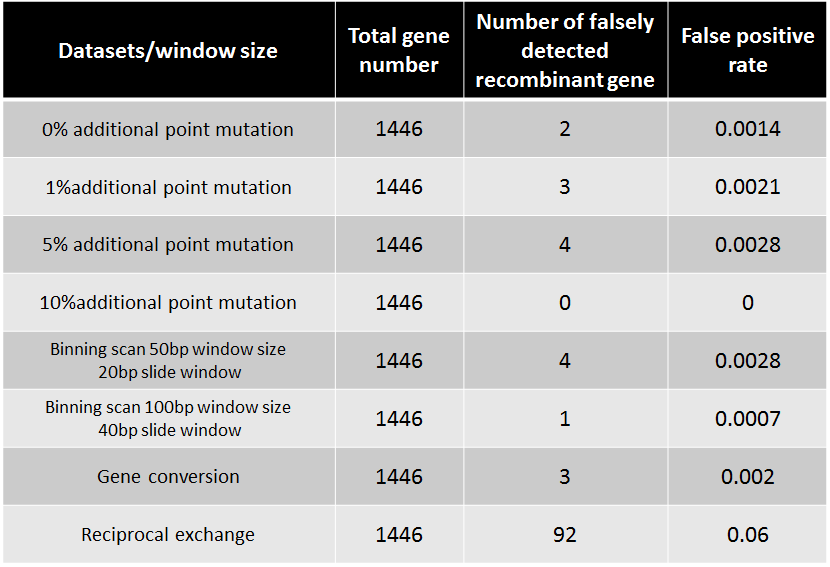


**b**


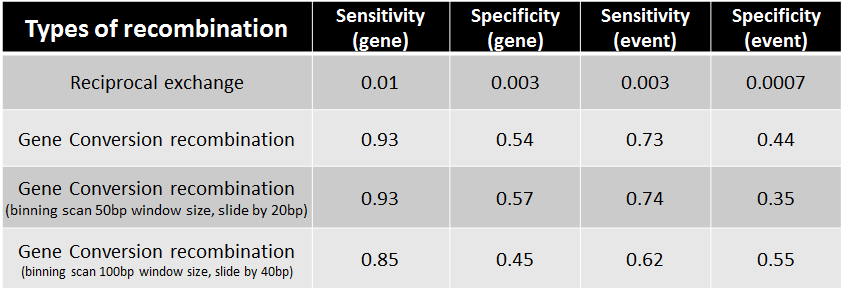

Supplement: Additional file 10: Table S4. — Summary of evaluation of the recombination detection pipeline with simulated data. a) false positive rates, sensitivity, and b) specificity of the recombination detection pipeline using simulated datasets of trans-sialidase gene family members, recombination positive data contains 200 recombinant genes with 1000 recombinant events. (DOCX 68 kb) [file 12864_2016_3037_MOESM10_ESM.docx]

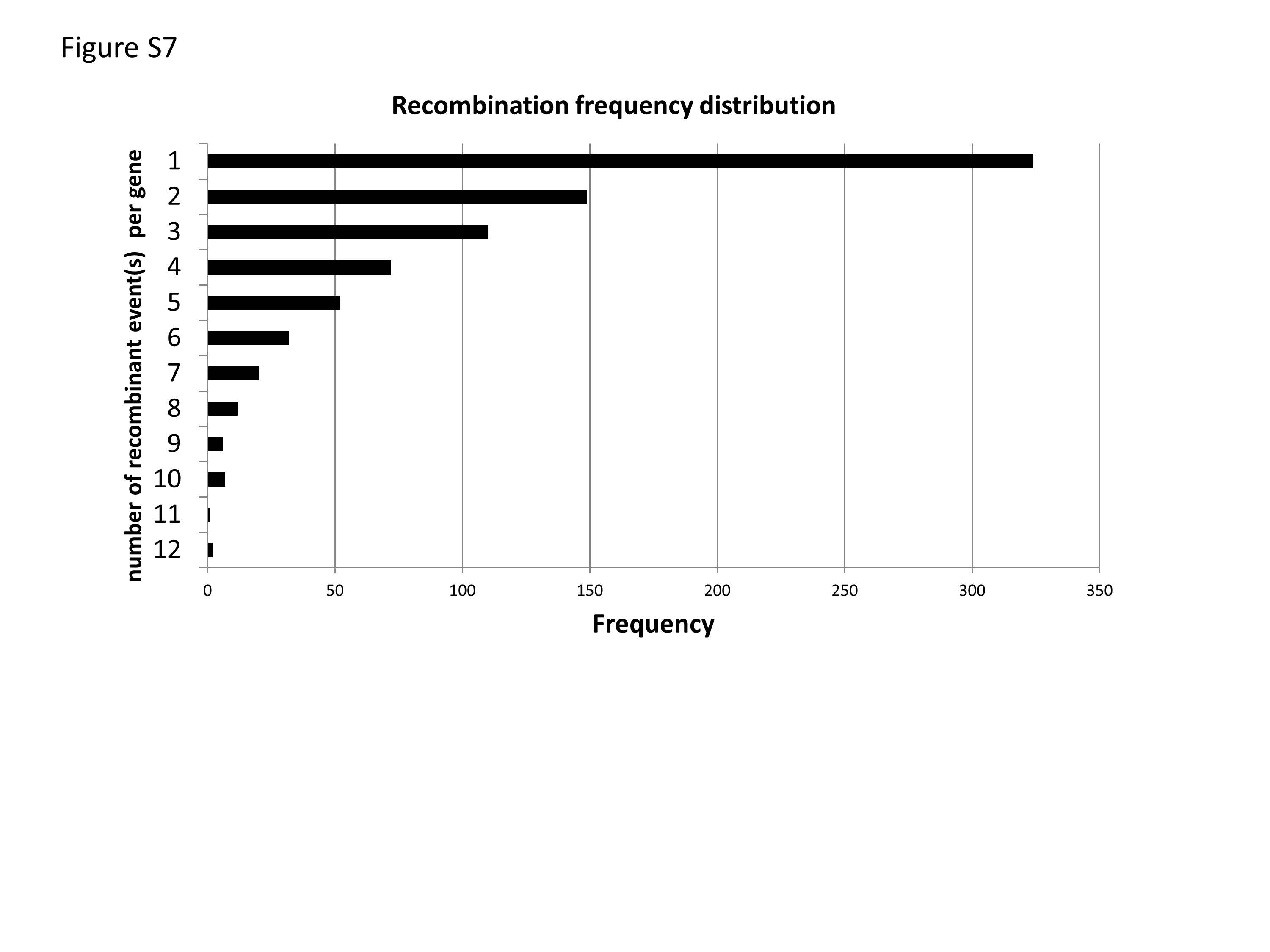

Supplement: Additional file 12: Figure S7. — Recombination frequency distribution. The distribution of recombination frequency summarized for each of all TcTs gene analyzed in this study. (TIF 521 kb) [file 12864_2016_3037_MOESM12_ESM.tif]

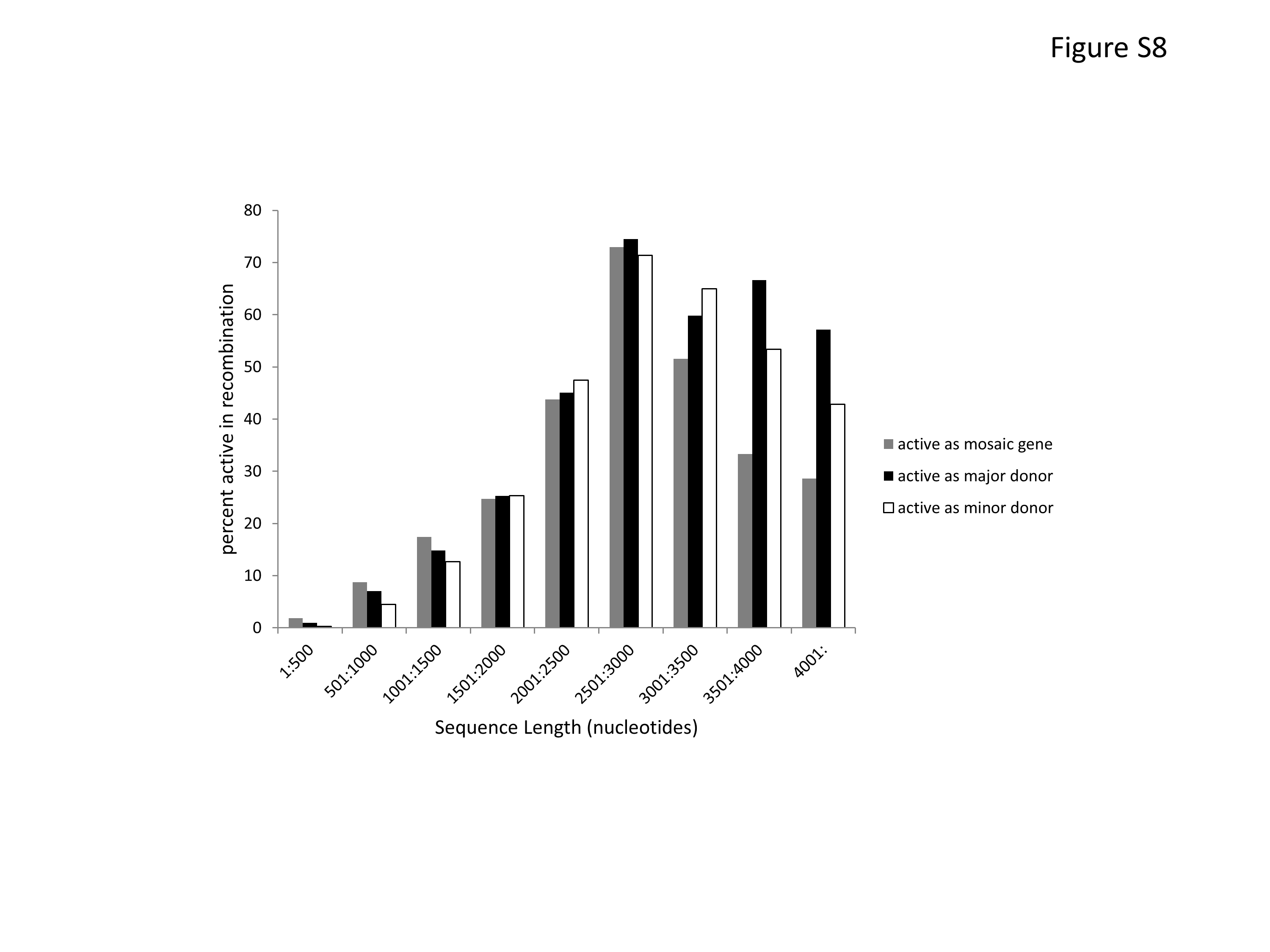

Supplement: Additional file 13: Figure S8. — TcTS genes participating in recombination in relation to gene size. The fraction of TcTS genes in each size class that served as a minor donor, major donor or are the mosiac product of recombination. Percent active as mosaic gene was calculated by dividing the number of mosaic gene by the total number of TcTS genes for each category; Percent active as major donor was calculated by dividing the number of genes that took role (at least once) as major donor by the total number of TcTS genes for each category; Percent active as minor donor was calculated by dividing the number of genes that took role (at least once) as minor donor by the total number of TcTS genes for each category. (TIF 565 kb) [file 12864_2016_3037_MOESM13_ESM.tif]
