# Supplementary material for: Recombination-driven generation of the largest pathogen repository of antigen variants in the protozoan Trypanosoma cruzi
Source: BMC Genomics. 2016 Sep 13;17(1):729. doi: 10.1186/s12864-016-3037-z (PMC5020489; doi:10.1186/s12864-016-3037-z)
Supplement: Additional file 14: Table S6. — Algorithms and respective parameters for the RDP package. (DOCX 17 kb) [file 12864_2016_3037_MOESM14_ESM.docx]

**Table S6: Algorithms and respective parameters for the RDP package**

| Algorithm name & reference | Parameters (that deviate from the default) |
| --- | --- |
| RDP [1] | window size=125 |
| GENECONV [2] | none |
| Chimaera [3] | variable sites per window=42 |
| MaxChi [4] | variable sites per window=42 |
| Bootscan [5] | window size=125, step size=20 |
| 3SEQ [6] | none |

1. Martin D, Rybicki E (2000) RDP: detection of recombination amongst aligned sequences. Bioinformatics 16: 562-563.

2. Padidam M, Sawyer S, Fauquet CM (1999) Possible emergence of new geminiviruses by frequent recombination. Virology 265: 218-225.

3. Posada D, Crandall KA (2001) Evaluation of methods for detecting recombination from DNA sequences: computer simulations. Proc Natl Acad Sci U S A 98: 13757-13762.

4. Smith JM (1992) Analyzing the mosaic structure of genes. J Mol Evol 34: 126-129.

5. Martin DP, Posada D, Crandall KA, Williamson C (2005) A modified bootscan algorithm for automated identification of recombinant sequences and recombination breakpoints. AIDS Res Hum Retroviruses 21: 98-102.

6. Boni MF, Posada D, Feldman MW (2007) An exact nonparametric method for inferring mosaic structure in sequence triplets. Genetics 176: 1035-1047.
